# Comparative interactomics analysis of different ALS-associated proteins identifies converging molecular pathways

**DOI:** 10.1007/s00401-016-1575-8

**Published:** 2016-05-10

**Authors:** Anna M. Blokhuis, Max Koppers, Ewout J. N. Groen, Dianne M. A. van den Heuvel, Stefano Dini Modigliani, Jasper J. Anink, Katsumi Fumoto, Femke van Diggelen, Anne Snelting, Peter Sodaar, Bert M. Verheijen, Jeroen A. A. Demmers, Jan H. Veldink, Eleonora Aronica, Irene Bozzoni, Jeroen den Hertog, Leonard H. van den Berg, R. Jeroen Pasterkamp

**Affiliations:** Department of Translational Neuroscience, Brain Center Rudolf Magnus, University Medical Center Utrecht, Utrecht, The Netherlands; Department of Neurology and Neurosurgery, Brain Center Rudolf Magnus, University Medical Center Utrecht, Utrecht, The Netherlands; Department of Biology and Biotechnology Charles Darwin and IBPM, Sapienza University of Rome, Rome, Italy; Center for Life Nano Science@Sapienza, Istituto Italiano di Tecnologia, Rome, Italy; Department of (Neuro)Pathology, Academic Medical Center, Amsterdam, The Netherlands; Swammerdam Institute for Life Sciences, Center for Neuroscience, University of Amsterdam, Amsterdam, The Netherlands; Proteomics Center, Erasmus University Medical Center, Rotterdam, The Netherlands; Hubrecht Institute-KNAW and University Medical Center Utrecht, Utrecht, The Netherlands; Centre for Integrative Physiology, The University of Edinburgh, Edinburgh, UK; Department of Molecular Biology and Biochemistry, Graduate School of Medicine, Osaka University, Osaka, Japan

**Keywords:** Amyotrophic lateral sclerosis, FMRP, FUS, C9orf72, Motor neuron, TDP-43

## Abstract

Amyotrophic lateral sclerosis (ALS) is a devastating neurological disease with no effective treatment available. An increasing number of genetic causes of ALS are being identified, but how these genetic defects lead to motor neuron degeneration and to which extent they affect common cellular pathways remains incompletely understood. To address these questions, we performed an interactomic analysis to identify binding partners of wild-type (WT) and ALS-associated mutant versions of ATXN2, C9orf72, FUS, OPTN, TDP-43 and UBQLN2 in neuronal cells. This analysis identified several known but also many novel binding partners of these proteins. Interactomes of WT and mutant ALS proteins were very similar except for OPTN and UBQLN2, in which mutations caused loss or gain of protein interactions. Several of the identified interactomes showed a high degree of overlap: shared binding partners of ATXN2, FUS and TDP-43 had roles in RNA metabolism; OPTN- and UBQLN2-interacting proteins were related to protein degradation and protein transport, and C9orf72 interactors function in mitochondria. To confirm that this overlap is important for ALS pathogenesis, we studied fragile X mental retardation protein (FMRP), one of the common interactors of ATXN2, FUS and TDP-43, in more detail in in vitro and in vivo model systems for FUS ALS. FMRP localized to mutant FUS-containing aggregates in spinal motor neurons and bound endogenous FUS in a direct and RNA-sensitive manner. Furthermore, defects in synaptic FMRP mRNA target expression, neuromuscular junction integrity, and motor behavior caused by mutant FUS in zebrafish embryos, could be rescued by exogenous FMRP expression. Together, these results show that interactomics analysis can provide crucial insight into ALS disease mechanisms and they link FMRP to motor neuron dysfunction caused by FUS mutations.

**Electronic supplementary material:**

The online version of this article (doi:10.1007/s00401-016-1575-8) contains supplementary material, which is available to authorized users.

## Introduction

Amyotrophic lateral sclerosis (ALS) is a neurodegenerative disease characterized by the loss of upper and lower motor neurons resulting in progressive muscle weakness and finally death due to respiratory failure. Median survival is 3–5 years after symptom onset and the only treatment available is riluzole, which increases life expectancy with on average 3 months. More effective therapeutic strategies for ALS are needed, but their design requires further insight into the pathogenic mechanisms underlying this disease.

Work during the past several years has significantly increased our understanding of the genetic basis of ALS and has led to the identification of disease-causing mutations in various genes including chromosome 9 open reading frame 72 (*C9orf72)*, fused in sarcoma (*FUS*), optineurin (*OPTN*), profilin 1 (*PFN1*), sequestosome 1 (*SQSTM1*), superoxide dismutase 1 (*SOD1*), TANK-binding kinase 1 (*TBK1*), TAR DNA-binding protein 43 (*TARDBP*), ubiquilin 2 (*UBQLN2*), vesicle-associated membrane protein-associated protein B and C (*VAPB*) and valosin-containing protein (*VCP)* [65]. In addition, defects in genes such as Ataxin-2 (*ATXN2)* and non-imprinted in Prader–Willi/Angelman syndrome 1 (*NIPA1*) were shown to affect disease susceptibility, onset or progression [4, 19]. These genetic findings have triggered the identification of cellular defects contributing to ALS pathogenesis, e.g., protein misfolding, mitochondrial dysfunction, oxidative stress, cytoskeletal alterations, and dysregulation of RNA metabolism [69]. A pathological hallmark of ALS is the presence of protein inclusions in degenerating motor neurons. The molecular signatures of these inclusions have in part been characterized [5] and show a remarkable overlap with genetic findings. For example, TDP-43, OPTN and UBQLN2 are common constituents of ALS-associated protein inclusions, while mutations in *TARDBP*, *OPTN* and *UBQLN2* occur in ALS patients [18, 53, 75]. However, while only a relatively small number of patients carry mutations in these genes, TDP-43 and other proteins localize to aggregates in a large number of patients [5]. These observations hint at a general role for these proteins in ALS pathogenesis. However, for many genetic defects and ALS-associated proteins it remains incompletely understood how they contribute to the development of motor neuron degeneration and ALS.

Recent studies show that identification of protein binding partners of proteins associated with ALS is a fruitful approach for characterization of the pathological mechanisms underlying this disease [21, 23, 28, 47, 52, 76, 90, 93]. For example, interactomics analyses have recently implicated survival motor neuron (SMN) and U1-snRNP in ALS pathogenesis associated with *FUS* mutations [28, 76, 90]. Interestingly, the few reported interactomes of ALS-associated proteins contain interactors that are known to harbor mutations in patients. For example, FUS interactomes reported so far contain heterogeneous nuclear ribonucleoprotein (hnRNP) A1 and hnRNPA2B1, while the genes encoding these proteins contain disease-causing mutations in a select group of patients [44]. This observation highlights a potential for interactome analysis to identify novel ALS-associated proteins. Further, it underlines the fact that these proteins participate in overlapping protein complexes and thus may affect related cellular processes. Such convergence at the molecular and cellular level is interesting from a therapeutic perspective as it may enable the design of therapies that target larger cohorts of patients.

To provide further insight into the pathogenic mechanisms underlying ALS and to determine the extent to which ALS-associated proteins share binding partners, we performed interactomics analyses for six ALS-associated proteins in neuronal cells: ATXN2, C9orf72, FUS, OPTN, TDP-43 and UBQLN2. Analyses were performed in parallel to minimize experimental variability. Because gene mutations may lead to gain- or loss-of-function phenotypes and changes in subcellular localization of the affected protein, interactomes were determined for wild-type proteins or proteins carrying an ALS-associated mutation (except C9orf72). Our study reveals a striking overlap between the interactomes of ATXN2, FUS, and TDP-43, and the interactomes of OPTN and UBQLN2, while interacting proteins for C9orf72 were distinct. Furthermore, mutations in OPTN and UBQLN2 altered interactome composition. Finally, in-depth characterization of one of the shared interactors of ATXN2, FUS, and TDP-43, identified a functional link between ALS mutant FUS and fragile X mental retardation protein (FMRP), a translational repressor that controls synaptic function. Our finding that FMRP rescues defects in neuromuscular junction (NMJ) morphology and aberrant motor behavior caused by mutant FUS in zebrafish together with recent work linking FMRP and TDP-43 [14] identifies FMRP as a shared component of the pathogenic mechanisms downstream of multiple ALS-associated proteins.

## Materials and methods

### Immunoprecipitation and immunoblotting

For the co-immunoprecipitation of endogenous protein complexes, N2A or NSC-34 cells from one 10-cm dish were lysed in 150 μl lysis buffer [20 mM Tris–HCl pH 7.5, 150 mM NaCl, 1 % NP-40, 10 % glycerol, complete protease inhibitor cocktail (Roche)], incubated on ice for 10 min and centrifuged at 13,200 rpm for 10 min at 4 °C. Lysates were incubated for 1 h at 4 °C using 1 μg antibodies. Detailed information on antibodies and plasmids used can be found in the Supplementary Material. Samples were incubated with protein A or protein G magnetic beads for 30 min followed by 4 washes in lysis buffer. Precipitated proteins were eluted by boiling in NuPage LDS sample buffer containing 10 mM DTT for 5 min at 90 °C. For RNA dependency experiments, lysates were first treated with RNAse A (Roche) for 30 min at 4 °C. Recombinant FUS (Origene) and FMRP (Abnova) proteins were mixed in buffer (10 % glycerol, 0.5 % NP40, 100 mM glycine, 25 mM Tris–HCl, pH 7.4) and incubated for 1 h with protein A agarose beads pre-incubated with rabbit anti-FUS antibody. Immunoprecipitation was performed as described above. For RNA dependency experiments, total RNA was isolated from HEK293 cells using the RNAeasy mini kit (Qiagen) according to the manufacturer’s instructions and added to the protein mixture at concentrations ranging from 0.1 to 62.5 ng/μl. For the generation of crude mitochondrial fractions, N2A cells were homogenized in homogenization buffer [0.25 M sucrose, 0.2 mM EDTA, 20 mM HEPES pH 7.4, complete protease inhibitor cocktail (Roche)] and a pellet containing crude mitochondria was obtained after differential centrifugation of the lysate.

Proteins were separated in a SDS-PAGE gel and transferred onto nitrocellulose membrane (Hybond-C Extra; Amersham). Membranes were incubated in blocking buffer (TBS, 0.05 % Tween and 5 % milk powder) for 30 min at room temperature (RT), followed by incubation with the appropriate antibody overnight at 4 °C. After several washes with TBS-T, membranes were incubated with appropriate peroxidase conjugated secondary antibodies in blocking buffer for one hour at RT followed by incubation with Super Signal West Dura Extended Duration Substrate (Pierce) and exposed to ECL films (Pierce). For quantification of Western blots, intensity measurements of the protein bands were performed using ImageJ.

### Biotin-streptavidin pull down

Immunoprecipitation of biotin-tagged proteins was performed as described previously [16, 28]. It is important to note that cell lysis could allow bait proteins to interact with proteins that they normally would not be able to bind (e.g., because of their localization in distinct cellular compartments). While this is an important point to consider, so far we have performed bioIPs for >40 different bait proteins (including cytoplasmic, nuclear or membrane-associated proteins) and never found strong evidence for such post-lysis artifacts.

### In-gel analysis and LC–MS/MS analysis

Samples were separated in a NuPAGE Novex 4–12 % Bis/Tris gradient gel following the manufacturer’s instructions. For silver staining, gels were fixed in 50 % methanol for 30 min, followed by incubation in 10 μM DTT for 15 min and incubation in 0.1 % (w/v) AgNO_3_ for 20 min. Subsequent gels were developed using 0.25 M anhydrous sodium carbonate containing 0.02 % (w/v) formaldehyde. For mass spectrometry analysis, gels were stained using GelCode blue stain reagent (Pierce). Mass spectrometry analysis of interacting proteins was done as described previously [96]. Raw data were analyzed by MaxQuant (version 1.4.1.2) [12] as described in the Supplementary Material. GO analysis was performed using PANTHER [57].

### Primary motor neuron culture, transfection and immunocytochemistry

All animal care and use was in line with institutional, national and European legislation. Mice (C57BL/6) were purchased from Charles River. Primary motor neurons were isolated from the ventral part of spinal cords of E13.5 mouse embryos and cultured as described in the Supplementary Material. After 48 h, cells were transfected using magnetic beads (Oz Biosciences) as described by Fallini et al. [20].

Primary motor neurons were fixed with 4 % PFA for 15 min at RT, permeabilized with 0.1 % Triton X-100 in PBS for 5 min at RT, blocked in PBS containing 2.5 % BSA, and incubated with primary antibodies in BSA supplemented with 5 % normal goat serum for 1 h at RT. After several washes in PBS, cells were incubated with a mixture of the appropriate Alexa Fluor^®^-labeled secondary antibodies (Life Technologies) for 1 h at RT. Then, cells were washed, counterstained with 4′,6′-diamidino-2-phenylindole (DAPI) (Sigma), washed extensively with PBS and mounted in DABCO.

### Immunohistochemistry on human spinal cord samples

Consent for autopsy was obtained in concordance with institutional regulations. Patient and control details are included in Supplementary Table S3. Double immunohistochemistry and double fluorescent-labeling were performed as described in the Supplementary Material. Due to antibody incompatibilities we were unable to perform double fluorescent-labeling for all interactors. Sections were analyzed using a laser scanning confocal microscope (Leica TCS Sp2, Wetzlar, Germany).

### Zebrafish studies

Zebrafish were kept and maintained under standard conditions. Injections were performed at the one-cell stage. FUS WT, FUS R521C and mCherry-FMRP were subcloned into a pCS2+ vector and mRNA was transcribed using the mMESSAGE Machine kit (Ambion) followed by phenol–chloroform purification and ethanol precipitation. mRNAs were diluted in RNAse free water (with 1 % phenol-red dye) at a concentration of 75–200 ng/μl and were pulse-injected using a pressure ejector. Touch-evoked escape response (TEER) was measured in developmentally normal zebrafish, as described previously [41].

For immunohistochemical analysis, zebrafish larvae were fixed in 4 % PFA overnight at 4 °C. Larvae were rinsed in PBS with 0.1 % tween (PBS-T) and then permeabilized with collagenase A (1 mg/ml) for 45 min at RT and stained consecutively with Alexa Fluor^®^ 488-conjugated alpha-bungarotoxin and anti-SV2 antibody as described in the Supplementary Material.

### Image analysis and statistics

For FMRP granule analysis, primary motor neurons were imaged using a Zeiss Axioskop2 microscope in combination with Axiovision SE64 software. For quantification of FMRP granules, an automated method in ImageJ was designed and statistical significance was tested using an ANOVA. For the zebrafish NMJ analysis, images were acquired using an Olympus Fluoview FV1000 confocal microscope and overlap between pre- and postsynapse was determined using the JACoP plugin for ImageJ [6] with Costes’ automatic thresholding.

### Synaptosome isolation

Zebrafish embryos were collected at 72 hpf and yolk sacs were removed by triturating in ice-cold Ringer’s salt solution (116 mM NaCl, 2.9 mM KCl, 1.8 mM CaCl_2_, 5 mM HEPES) with 50 mM EDTA. Then, 200–500 embryos were homogenized per condition in homogenization buffer (320 mM sucrose, 4 mM HEPES supplemented with protease inhibitors) and synaptosomes were obtained after differential centrifugation of the lysate.

### Quantitative RT-PCR

Zebrafish embryos were collected as described above and 150–200 embryos per condition were homogenized and fractionated in homogenization buffer supplemented with 2 % β-mercaptoethanol. TRIzol reagent was added to each fraction and total RNA was isolated. After DNAse treatment, cDNA was synthesized and quantitative real-time PCR was performed using primers listed in the Supplementary Materials. Fractions were normalized to their respective inputs and fold changes were then calculated compared to non-injected controls using standard comparative ΔΔCT methods. Samples from three experiments were amplified in triplicate and results were statically analyzed using Student’s *t* test.

### Polysomal fractionations

Polysomal fractionations from N2A cells were performed using a protocol adapted from Gismondi et al. [27].

## Results

### Interactomics analysis of ALS-associated proteins

Identification of protein binding partners of proteins associated with ALS can aid the characterization of the pathogenic mechanisms underlying this disease [21, 23, 28, 52, 76, 77, 90]. To further dissect ALS disease mechanisms and to possibly unveil common disease pathways downstream of multiple ALS-associated proteins, we used a biotin-streptavidin pull down system [16, 28] in combination with mass spectrometry to determine the interactomes of six ALS-associated proteins. ATXN2, C9orf72, FUS, OPTN, TDP-43 and UBQLN2 were selected on basis of genetic and pathological data. These proteins are the focus of many current studies on ALS pathogenesis, are localized to ALS-associated inclusions in a large proportion of patients [5, 43], and/or are poorly characterized at the functional level (C9orf72).

**Fig. 1:**
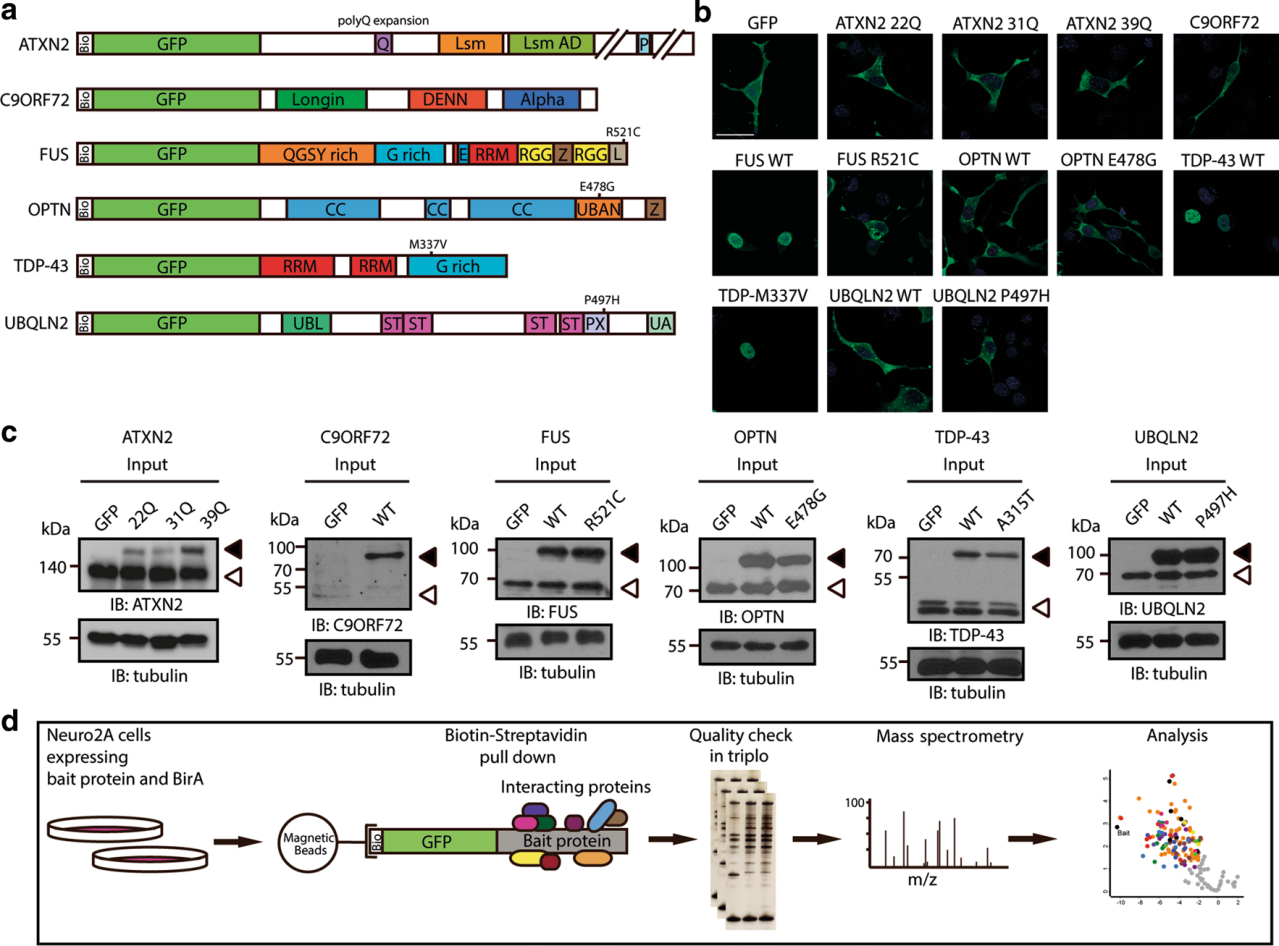
Experimental setup of interactomics analysis of six ALS-associated proteins. **a** Schematic representation of bioGFP-tagged ATNX2, C9orf72, FUS, OPTN, UBQLN2 and TDP-43. *Numbers* indicate common ALS-associated mutations. *Bio* biotin-tag, *CC* coiled-coil domain, *DENN* differentially expressed in normal and neoplasia, *E* nuclear export signal, *G-rich* glycine-rich region, *L* nuclear localization signal, *Lsm* like-Sm domain, *Lsm AD* Lsm associated domain, *P* Poly(A)-binding protein interacting motif, *PX* proline XX repeat region, *Q* poly-glutamine stretch, *QGSY* glutamine/glycine/serine/tyrosine-rich region, *RRM* RNA recognition motif, *RGG* arganine/glycine-rich region, *ST* heat shock chaperone binding motif, *UBAN* ubiquitin binding in ABIN and NEMO domain, *UBL* ubiquitin-like domain, *UA* ubiquitin-associated domain, *WT* wild-type, *Z* zinc finger motif. **b** Immunocytochemistry for GFP on Neuro2A (N2A) cells transfected with the indicated constructs (shown in **a**). Exogenous proteins show an endogenous distribution pattern. **c** Western blot analysis of lysates of N2A cells transfected with the indicated constructs. Tubulin is used a loading control. Exogenous proteins are indicated with *closed arrowheads*, *open arrowheads* indicate endogenous proteins. **d** Overview of experimental design. N2A cells were co-transfected with bioGFP-tagged constructs and the biotin ligase BirA. After 48 h, exogenous proteins were purified using magnetic streptavidin-coated beads. Purified protein complexes were separated using gel electrophoresis and subjected to silver staining to confirm equal protein loading and detection of bait and interacting proteins. Mass spectrometry analysis was performed, followed by MaxQuant analysis and statistical analysis using PERSEUS. *IB* immunoblot. *Scale bar* 30 μm

To identify interactors, biotin-GFP-tagged (bioGFP) constructs were generated for ATXN2, C9orf72, FUS, OPTN, TDP-43, and UBQLN2 as well as for a common ALS-associated mutant variant of each of these proteins (ATXN2 31Q, 39Q, FUS R521C, OPTN E478G, TDP-43 M337V and UBQLN2 P497H) (Fig. 1a). Hexanucleotide repeat expansions in C9orf72 cause ALS but these expansions are found in the non-coding part of the gene and are therefore not present in C9orf72 protein [17, 68]. Nevertheless, C9orf72 was included to further define its functional role(s). Neuronal cells (N2A) were co-transfected with plasmids encoding the bacterial biotin ligase BirA and one of the bioGFP-tagged constructs. Importantly, the subcellular localization of transfected epitope-tagged proteins was indistinguishable from the reported distribution of their wild-type or ALS-mutated endogenous counterparts (Fig. 1b). Furthermore, immunoblotting showed that expression of epitope-tagged ATXN2, FUS, OPTN, and TDP-43 was equivalent to or lower than endogenous proteins, while exogenous UBQLN2 was expressed at slightly higher levels. Endogenous expression of C9orf72 was low in N2A cells consistent with weak expression of C9orf72 in other cell types [33, 83] (Fig. 1c). Biotinylated bait proteins and their binding partners were captured from N2A cell lysates with magnetic streptavidin beads, separated on gel and subjected to immunoblotting with anti-GFP antibodies or silver staining to confirm pull down efficiency (Figs. 1d, S1, S2). Following this confirmation, samples were subjected to mass spectrometry analysis. Identification and quantification of proteins was carried out using MaxQuant software [11] and statistical analyses were performed using the open PERSEUS environment. For pairwise comparison of GFP and WT interactomes, and WT and mutant interactomes, Welch’s *t* test statistics were applied with an FDR of 0.01 and S0 of 1.5 (the S0 parameter sets a threshold for minimum fold change). With these criteria, 163 interactors of ATXN2, 53 interactors of C9orf72, 123 interactors of FUS, 33 interactors of OPTN, 140 interactors of TDP-43, and 104 interactors of UBQLN2 were identified. These interactors included both known and novel binding partners of the different ALS-associated protein baits. It is important to note that some of the identified interactors may engage in indirect interactions that require other proteins for binding to the selected bait proteins. Below each of the interactomes identified in this study is discussed in more detail.

#### ATXN2

**Fig. 2:**
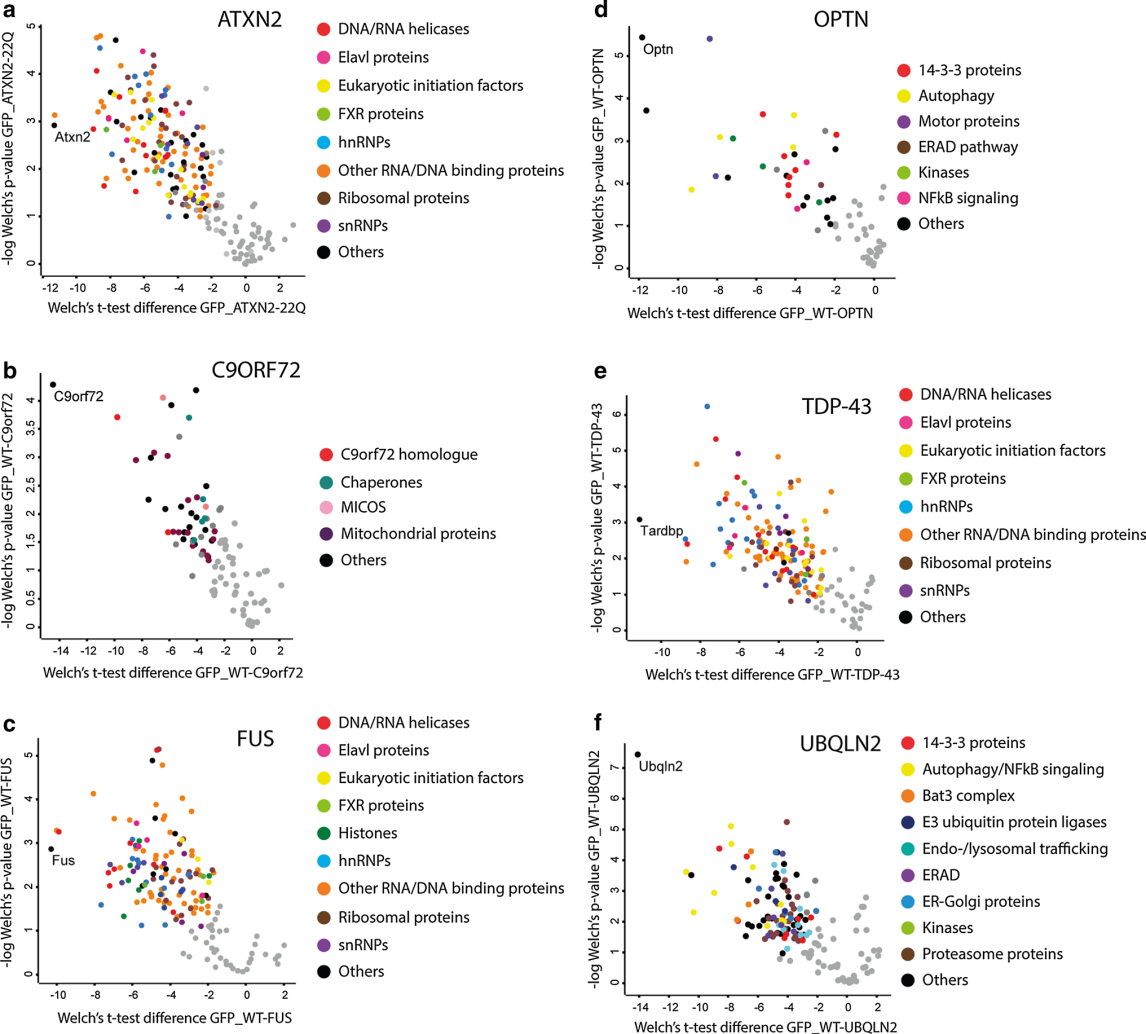
Quantitative analysis of the binding partners of six wild-type ALS-associated proteins. **a**–**f** Volcano plots showing interactors of wild-type, non-mutated forms of ATXN2 22Q (**a**), C9ORF72 (**b**), FUS (**c**), OPTN (**d**), TDP-43 (**e**) and UBQLN2 (**f**). Logarithmized ratios of relative protein intensities are plotted against the negative logarithmic p value from triplicate experiments (Welch’s *t* test; S0: 1.5, FDR: 0.01). PERSEUS was used for statistical analysis and visualization; *colored dots* represent significantly enriched proteins and the *color* refers to the corresponding functional category indicated at the *right*. *FXR* fragile-X related, *MICOS* mitochondrial contact site complex, *ERAD* endoplasmatic-reticulum-associated degradation

ATXN2 is an RNA-binding protein that is involved in polyadenylation, translation, stress granule assembly and receptor endocytosis [79]. Binding partners of ATXN2 identified in our study included the known interactors polyadenylate-binding protein 1 (Pabpc), probable ATP-dependent RNA helicase DDX6 (Ddx6) [55, 63], and argonaute-2/Eif2c2 [55] and a range of other proteins involved in RNA metabolism, including: hnRNPs, eukaryotic initiation factors, RNA helicases and splicing factors (Fig. 2a; Supplementary Table S1a). GO analysis indicated that ATXN2 binds proteins known to be part of the ribonucleoprotein (RNP) complex, stress granules, eIF3 complex and the spliceosome and are associated with cellular processes including mRNA translation, mRNA metabolic processing, splicing, posttranscriptional regulation and RNA localization (Supplementary Table S1a). The interactors hnRNPA1, hnRNPA2B1, Matr3, FMRP and Prkra have previously been associated with neurodegenerative diseases (ALS, multisystem proteinopathy, fragile X-associated tremor/ataxia syndrome (FXTAS), fragile-X syndrome (FXS) and dystonia parkisonism).

#### C9orf72

The function of C9orf72 is largely unknown although homology searches identified a putative DENN domain [50, 92]. Intriguingly, the top hit of our interactomics analysis was Smith–Magenis syndrome chromosome region, candidate 8 (Smcr8), another DENN domain containing protein [92]. In addition, we identified a plethora of mitochondrial proteins including WD repeat containing protein 41 (Wdr41), Mitochondrial import inner membrane translocase subunit TIM50 (Timm50), Inner membrane protein mitochondrial (Immt), solute carrier family (Slc)25a3, Slc25a12, Slc25a5, Slc16a1, Slc25a4, ATPase family AAA-domain containing protein 3 (Atad3) and voltage-dependent anion-selective channel protein 3 (Vdac3) (Fig. 2b; Supplementary Table S1b). This suggests a role for C9orf72 in mitochondrial function. In line with this observation, GO analysis showed enrichment for proteins present in the inner mitochondrial membrane and Western blotting detected C9orf72 in mitochondria-enriched fractions (Fig. S3; Supplementary Table S1b).

#### FUS

FUS (also known as translated in liposarcoma (TLS)) is an hnRNP protein that has been associated with multiple steps in RNA metabolism. A large number of ALS-associated mutations in *FUS* target its nuclear localization signal (NLS) and thereby cause cytosolic mislocalization [48, 81]. However, FUS also localizes to cytosolic aggregates in motor neurons of ALS patients that do not carry mutations in *FUS* [5]. Many of the proteins found in bioGFP-FUS protein complexes were RNA/DNA-binding proteins and GO analysis of all proteins found to interact with FUS showed a strong enrichment for categories such as RNA processing and splicing, mRNA metabolic process, DNA repair, RNA transport, RNA stability and translation. The cellular compartments linked to the FUS interactome were RNP complex, spliceosome and ribosome (Fig. 2c; Supplementary Table S1c). FUS interactors identified in this study included previously characterized binding partners of FUS such as polypyrimidine tract-binding protein (Ptbp1) and serine and arginine-rich (SR) proteins [56]. Furthermore, the FUS interactome identified here showed a considerable overlap with previously reported FUS interactomes; e.g., 45 % of the interactors identified in our study were also found by Sun et al. [28, 76, 90]. This is significant considering the fact that these studies used different methods and cell types to identify interactors. Finally, FUS pull down led to the identification of several interactors with known roles in motor neuron disease or other neurodegenerative disorders, including hnRNPA1, hnRNPA2B1, Matrin3 and FMRP [3, 38, 44].

#### OPTN

Mutations in OPTN were already known to cause primary open-angle glaucoma when it was found that both dominant and recessive mutations in OPTN cause ALS [53]. OPTN is an inhibitor of NF-κB signaling [97], an autophagy receptor [85] and a regulator of vesicular transport associated with the Golgi apparatus [73]. Among the interactors of OPTN were the known interactors Ubiquitin (Ub), Tbk1, Tax1-binding protein 1 (Tax1bp1), and Sqstm1 [9, 39, 85]. In addition, other known regulators of NF-κB signaling and autophagy like TNFAIP3-interacting protein 1 (Tnip1), E3 ubiquitin-protein ligase RNF31 (Rnf31) and Vcp were detected (Fig. 2d; Supplementary Table S1d). Furthermore, different members of the Nuclear protein localization protein 4 homolog (Nploc4)-Vcp-Ubiquitin fusion degradation protein 1 homolog (Ufd1l) complex were present in the OPTN interactome indicating a role for OPTN in the Endoplasmic-Reticulum Associated Protein Degradation (ERAD) pathway. The OPTN interactome also included the linear ubiquitin chain assembly complex (LUBAC) that has recently been implicated in ubiquitin-signaling. LUBAC links K63-linear ubiquitin chains to specific substrates, e.g. the receptor interacting protein (RIP), thereby regulating signaling cascades including NF-κB-signaling. OPTN competes with NF-κB essential molecule (NEMO) for binding polyubiquitinated RIP, thereby inhibiting NF-κB signaling. GO analysis revealed that OPTN interactors are involved in protein localization and intracellular transport (Supplementary Table S1d). Interestingly, three of the identified interactors, Vcp, Sqstm1 and Tbk1, have recently been implicated in ALS [9, 22, 25, 37].

#### TDP-43

TDP-43 was first identified as a major constituent of ALS cytoplasmic inclusions [62] and subsequent studies showed that mutations in the gene encoding TDP-43, *TARDBP*, segregate with disease in familial ALS [75]. TDP-43 is a DNA and RNA-binding protein that plays an important role in transcription and splicing. ALS-associated mutations in *TARDBP* cluster in its C-terminal glycine-rich domain. As reported for FUS, many DNA- and RNA-binding proteins were detected in the interactome of TDP-43 (Fig. 2e; Supplementary Table S1e) and GO analysis revealed that these proteins play a role in RNA processing, gene expression, RNA splicing, posttranscriptional regulation of gene expression and translation. Two earlier studies investigated interacting proteins of TDP-43 [23, 52] and these previously reported interactomes display considerable overlap with the one reported in our study (60 % of the interactors identified in our TDP-43 pull down were also identified by Freibaum et al. [23] and 60 % of the interactors identified by Ling et al. [52] were also found by us). A number of validated TDP-43 binding partners were present in our screen including hnRNPA2B1 and hnRNPA1 [7, 70], hnRNPH, hnRNPK, hnRNPQ (Syncrip), interleukin enhancer binding factor 3 (Ilf3) [23, 52], Matrin3 [38], Pabpc [23] and probable ATP-dependent RNA helicase DDX5 (Ddx5) [42]. In addition, several of the identified proteins had previously been linked to neurodegenerative diseases like hnRNPA1, hnRNPA2B1, Matrin3 and FMRP.

#### UBQLN2

Ubiquilins form a functional link between ubiquitination machinery and the proteasome [45]. Indeed, many proteasome subunits were found in the UBQLN2 interactome, as well as two ubiquitin ligases [E3 ubiquitin-protein ligase TRIM32 (Trim32) and E3 ubiquitin-protein ligase Itchy (Itch)] and many proteins related to the protein degradation pathway including ubiquilin 1 (Ubqln1), Optn, Sqstm1, Vcp, ubiquitin fusion degradation protein 1 homolog (Ufd1l) and Tbk1 (Fig. 2f; Supplementary Table S1f). As mentioned above, Sqstm1, Vcp and Tbk1 have been associated with ALS, suggesting that these proteins are part of a common complex and may cause ALS by disruption of a common cellular mechanism. Interestingly, Ataxin-10 (Atxn10) and Huntingtin-associated protein 1 (Hap1), proteins associated with the neurodegenerative disease spinocerebellar ataxia 10 (SCA10) and Huntington’s disease, respectively, were identified as UBQLN2 interactors. GO analysis indicated that the UBQLN2 interactome is enriched for proteins involved in protein catabolic process, the ERAD pathway and protein localization (Supplementary Table S1f).

### ALS-associated mutations alter composition of OPTN and UBQLN2 interactomes

**Fig. 3:**
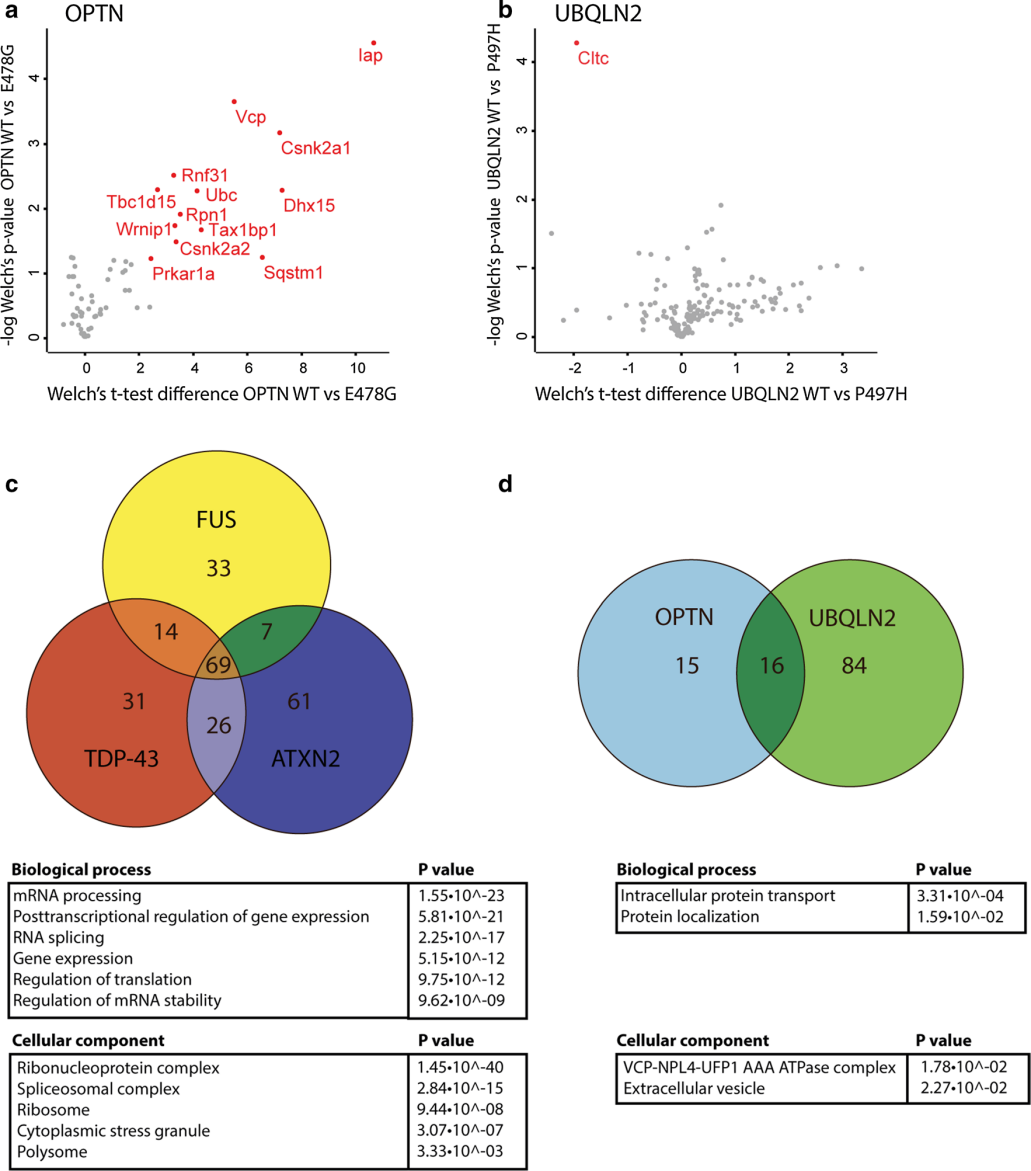
The effect of ALS-associated mutations on interactome composition and overlap between different interactomes. **a**, **b** Volcano plots showing a comparison of the interactors of OPTN E478G and OPTN wild-type (WT) (**a**) and UBQLN2 P487H and UBQLN WT (**b**). Logarithmized ratios of relative protein intensities are plotted against the negative logarithmic *p* value from triplicate experiments (Welch’s *t* test; S0: 1.5, FDR: 0.01). PERSEUS was used for statistical analysis and visualization; *red dots* and *labels* refer to proteins significantly altered compared to the WT interactome. **c**, **d** Venn diagrams showing the number of unique and overlapping interactors of ATXN2, FUS and TDP-43 (**c**) and of OPTN and UBQLN2 (**d**). *Tables* show GO analyses performed on the shared interactors. *Csnk2a* casein kinase II subunit alpha, *Cltc* clathrin heavy chain 1, *Dhx15* Pre-mRNA-splicing factor ATP-dependent RNA helicase DHX15, *Iap* IgE-binding protein, *Prkar1a* cAMP-dependent protein kinase type I-alpha regulatory subunit, *Rnf31* E3 ubiquitin-protein ligase RNF31, *Rpn1*, Dolichyl-diphospho-oligosaccharideprotein glycosyltransferase subunit 1, *Sqstm1* Sequestosome1, *Tax1bp1* Tax1-binding protein 1, *Tbc1d15* TBC1 domain family member 15, *Ub* ubiquitin, *Vcp* valosin-containing protein, *Wrnip1* ATPase WRNIP1

Mutations may not only cause protein mislocalization or affect enzymatic activity but could also influence protein–protein interactions. To determine whether the ALS-associated mutations selected here influence protein–protein interactions, we compared the interactomes of wild-type and mutant proteins. No statistically significant differences were detected between the interactomes of wild-type and mutant ATXN2, FUS and TDP-43. However, OPTN and UBQLN2 wild-type and mutant proteins showed altered protein interaction patterns (Fig. 3a, b). This difference was most prominent for mutant OPTN, where interactions with 13 proteins were detected for OPTN WT but not OPTN E478G. These interactors were Casein kinase II subunit alpha (Csnk2a1, Csnk2a2), Pre-mRNA-splicing factor ATP-dependent RNA helicase DHX15 (Dhx15), IgE-binding protein (Iap), cAMP-dependent protein kinase type I-alpha regulatory subunit (Prkar1a), Rnf31, Dolichyl-diphospho-oligosaccharideprotein glycosyltransferase subunit 1 (Rpn1), Sqstm1, Tax1bp1, TBC1 domain family member 15 (Tbc1d15), Ub, Vcp and ATPase WRNIP1 (Wrnip1) (Fig. 3a). Other interactors that were specific for wild-type OPTN but that did not pass our selection criteria were Protein 2410002F23Rik, BAG family molecular chaperone regulator 2 (Bag2), DnaJ homolog subfamily B member 6 (Dnajb6), FAS-associated factor 2 (Faf2), Flotillin-1 (Flot1), Flotillin-2 (Flot2), Pericentriolar material 1 protein (Pcm1), Peptidyl-prolyl cis-trans isomerase A (Ppia), Proteasome subunit alpha type-7 (Psma7), Ras-related protein Rab-1A (Rab1a), RanBP-type and C3HC4-type zinc finger-containing protein 1 (Rbck1), Sphingosine-1-phosphate lyase 1 (Sgpl1), Store-operated calcium entry-associated regulatory factor (Tmem66) and Ufd11. The proteins that failed to bind mutant OPTN function in ubiquitin-dependent protein degradation and ER transport.

UBQLN2 P497H showed increased binding affinity for clathrin heavy chain 1 (Cltc) compared to UBQLN2 WT (Fig. 3b). Clathrin is a major component of clathrin-coated pits (CCPs) and vesicles involved in intracellular trafficking and receptor endocytosis. In summary, our pull down experiments do not reveal dramatic changes in protein–protein interactions caused by ALS-associated mutations, with the exception of OPTN E478G.

### Comparison between different interactomes reveals distinct and shared pathways

The experimental design of our study, i.e., parallel analysis of six ALS-associated proteins using an identical biochemical workflow, provided a unique opportunity to investigate potential overlap between ALS-associated interactomes. Comparison of the different wild-type interactomes identified high overlap between ATXN2, FUS and TDP-43 interactomes and a considerable overlap between the interactomes of OPTN and UBQLN2 (Fig. 3c, d). In contrast, the C9orf72 interactome did not overlap with any of the other interactomes. ATXN2, FUS and TDP-43 shared 69 interactors (Supplementary Table S2). These included RNA helicases, eIFs, Elavl proteins, hnRNPs, ribosomal subunits, components of the exon junction complex, spliceosome and other RNA-binding proteins. GO analysis of the shared proteins identified processes such as RNA processing, posttranscriptional regulation of gene expression, splicing and gene expression, which is in line with the reported functions of ATXN2, FUS and TDP-43 (Fig. 3c). The overlap between OPTN and UBQLN2 included Sqstm1, Vcp, Ubc, Tbk1 and Tax1bp1. These shared interactors have reported roles in autophagy and NF-κB signaling (Fig. 3d). Thus, ATXN2-FUS-TDP-43 and OPTN-UBQLN2 share interactors that are part of common cellular protein complexes and are likely to affect similar cellular processes.

### Common interactors of ATXN2, FUS and TDP-43 localize to mutant FUS aggregates

**Fig. 4:**
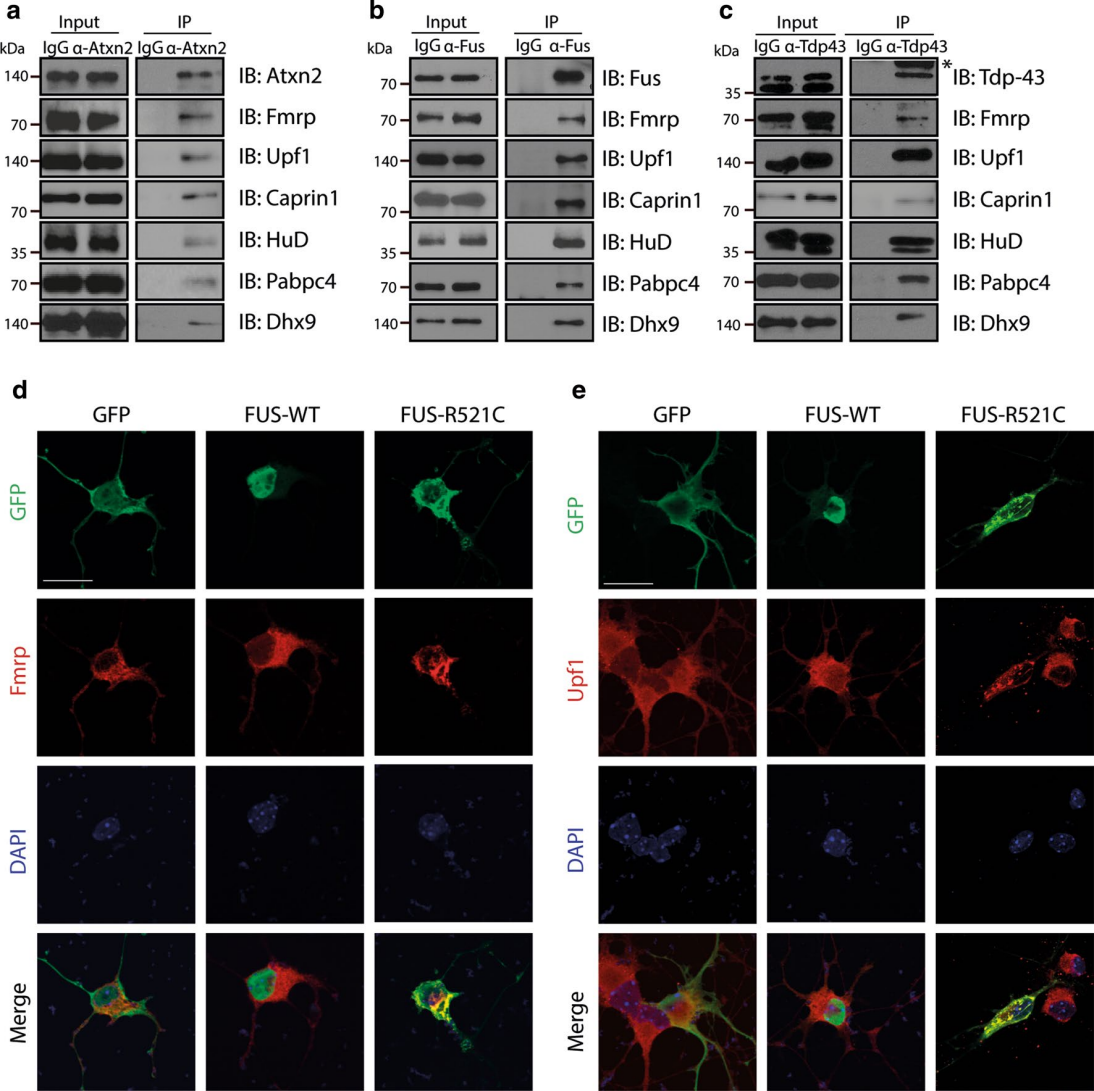
Interactors show endogenous binding to Atxn2, Fus and Tdp-43 and cytoplasmic mislocalization following transfection of mutant FUS. **a**–**c** Western blot analysis of input and immunoprecipitation (IP) samples with the indicated antibodies following endogenous immunoprecipitation with antibodies against Atxn2 (**a**), Fus (**b**), or Tdp-43 (**c**) from Neuro2A (N2A) cell lysates. *Asterisk* indicates IgG band. *IgG* IgG control antibody. **d**, **e** Dissociated primary motor neuron cultures generated from E13.5 mouse embryos were transfected with DNA constructs expressing GFP, FUS wild-type (WT) or FUS R521C and, after 2 days in culture, fixed and immunostained using antibodies against FMRP (**d**) or Upf1 (**e**). DAPI staining was used to visualize the cell nucleus. *IB* immunoblot. *Scale bar* 30 μm (**d**, **e**)

**Fig. 5:**
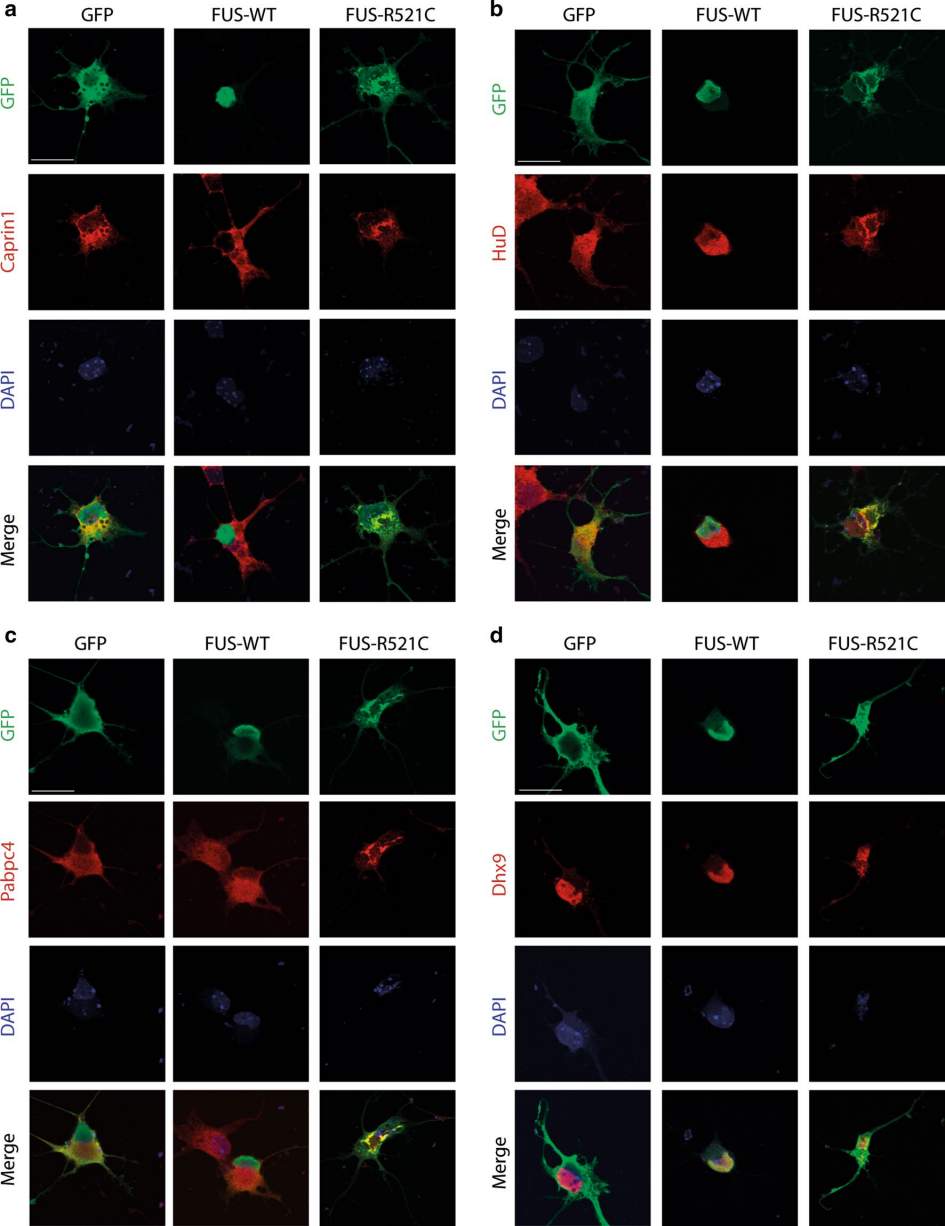
Interactors show cytoplasmic mislocalization following transfection of mutant FUS (continued). **a**–**d** Dissociated primary motor neuron cultures generated from E13.5 mouse embryos were transfected with DNA constructs expressing GFP, FUS wild-type (WT) or FUS R521C and, after 2 days in culture, fixed and immunostained using antibodies against Caprin1 (**a**), HuD (**b**), Pabpc4 (**c**) or Dhx9 (**d**). DAPI staining was used to visualize the cell nucleus. *Scale bar* 30 μm (**a**–**d**)

Molecular pathways shared by different ALS-associated proteins provide an opportunity to understand and treat ALS pathogenesis in larger groups of patients. To exploit this opportunity, we selected six interactors shared by ATXN2, FUS and TDP-43 on the basis of interactome abundance scores, literature and availability of experimental tools (FMRP, Upf1, Caprin1, HuD (Elavl4), Pabpc4, and Dhx9). First, co-immunoprecipitation was used to confirm the binding of these interactors to endogenous ATXN2, FUS and TDP-43 (Fig. 4a–c). One model to explain how ALS mutations cause neuron degeneration is that mutant proteins sequester their interactors into aggregates thereby causing their depletion and defects in specific cellular processes (e.g. [28, 60]). Therefore, we next determined whether Fmrp, Upf1, Caprin1, HuD, Pabpc4 or Dhx9 show an altered distribution in the presence of mutant FUS. Several ALS-associated mutations in FUS cause robust cytosolic mislocalization and formation of inclusions, in experimental settings or human motor neurons, respectively (e.g. [28, 29, 31, 48, 81, 82, 91]). GFP, wild-type FUS or FUS R521C were transfected in primary mouse motor neurons and subjected to immunocytochemistry for FUS and the different interactors at 2 days in vitro (DIV2). Following transfection of FUS R521C condensed accumulations of mutant FUS protein were detected in the cytoplasm of motor neurons that were clearly distinct from the weak, homogeneous cytoplasmic distribution observed for wild-type FUS. In line with their ability to bind FUS, all interactors were detected in mutant FUS cytoplasmic accumulations in primary motor neurons and showed a change in their cytoplasmic distribution compared to wild-type FUS neurons (Figs. 4d, e, 5). To assess whether a similar colocalization occurs in ALS patients, we performed immunohistochemistry for FUS and each of the six interactors on spinal cord sections from two patients harboring a FUS mutation (R521C) (Supplementary Table S3). Expression of FMRP, UPF1, CAPRIN1 and HUD, but not of PABPC4 and DHX9, was observed in mutant FUS inclusions in spinal motor neurons (Fig. S4; Supplementary Table S4) (70–85 % of FUS inclusions displayed immunolabeling for interactors). Because we confirmed FMRP, UPF1, CAPRIN1 and HUD as shared interactors of FUS and TDP-43 (Fig. 4b, c), we next asked whether these proteins also localize to TDP-43-positive inclusions. Indeed, CAPRIN1, FMRP and HUD were detected in phospho-TDP-43 inclusions in spinal motor neurons in ALS patients (Fig. S5, S6; Supplementary Table S3) (80–100 % of pTDP-43 inclusions displayed immunolabeling for interactors). Thus, FMRP, Caprin1 and HuD bind endogenous FUS and TDP-43, and colocalize with mutant FUS and TDP-43 in aggregate-like structures in spinal motor neurons. Of these interactors, FMRP and HuD have been implicated recently in TDP-43-induced motor neuron degeneration [2, 14, 20, 34]. To examine whether these proteins are indeed components of shared pathways downstream of multiple ALS-associated proteins and to further characterize their role in ALS pathogenesis, we selected one of these candidates, FMRP, for further functional studies in relation to FUS mutations.

### FUS-FMRP binding is direct and regulated by RNA

FMRP is encoded by the *FMR1* gene. Expansions of more than 200 CGG repeats in the 5′UTR of *FMR1* cause FXS and an intermediate repeat length of 50–200 repeats causes FXTAS, a late-onset neurodegenerative disorder. FMRP is a translational repressor with many different mRNA targets in neurons and plays an important role in synapse formation, including NMJ development and plasticity [95].

**Fig. 6:**
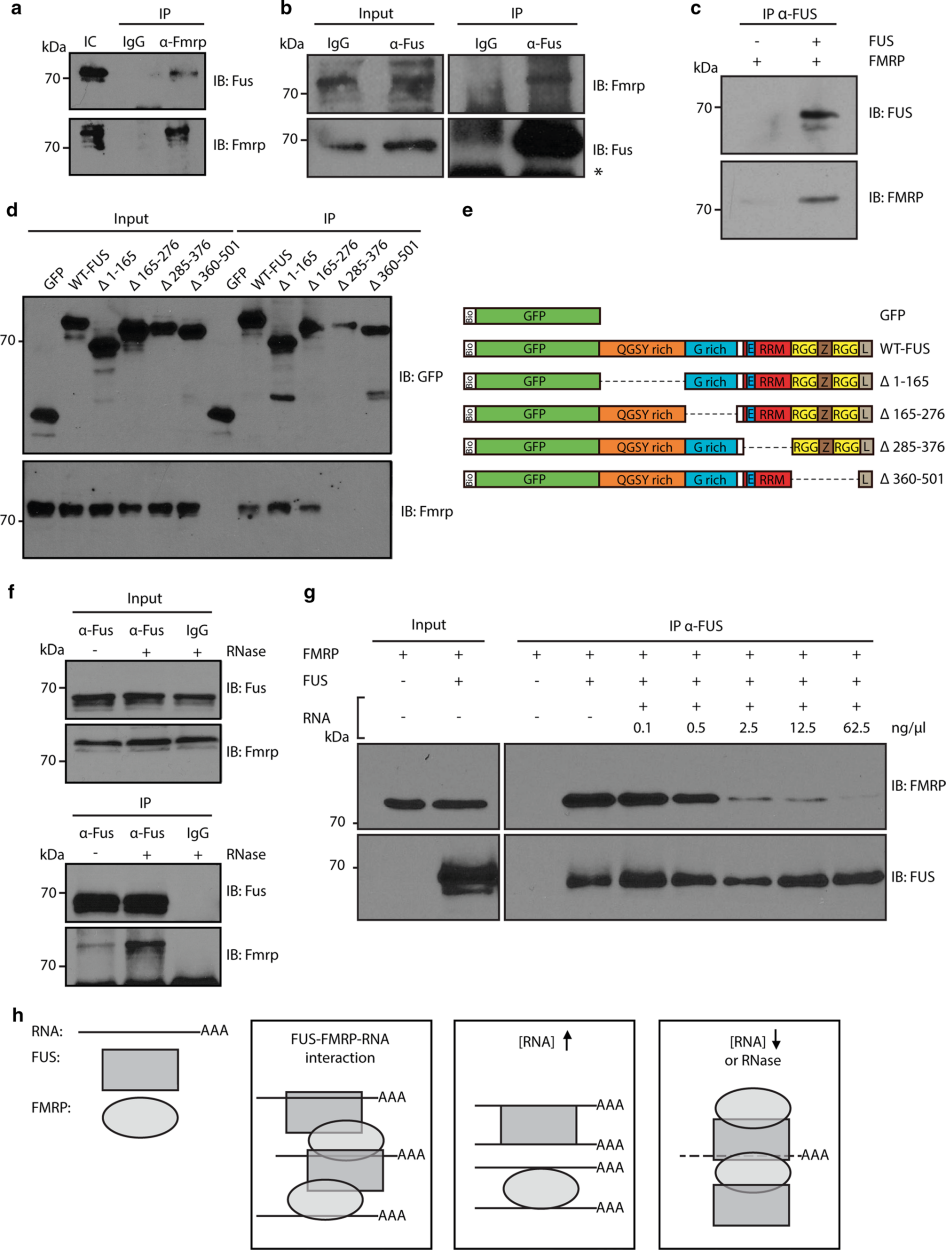
Biochemical characterization of FMRP-FUS binding. **a** FMRP pull down samples (IP) from NSC-34 cells were immunoblotted with the indicated antibodies. IgG was used as a negative control. **b** IP of Fus from E14 primary mouse cortical neurons. Input and IP samples were analyzed with the indicated antibodies. *Asterisk* indicates IgG band. **c** Recombinant FUS and FMRP proteins were mixed and immunoprecipitated with anti-FUS antibodies. Samples were analyzed with the indicated antibodies. **d** Neuro2A (N2A) cells were transfected with bioGFP-tagged FUS deletion constructs (indicated in **e**) and immunoprecipitated using streptavidin beads (IP). Samples were immunoblotted and probed with the indicated antibodies. **e** Schematic overview of bioGFP-tagged FUS deletion constructs used in **d**. *Bio* biotin-tag, *E* nuclear export signal, *G rich* glycine-rich domain, *L* nuclear localization signal, *RRM* RNA recognition motif, *RGG* arganine/glycine-rich region, *QGSY* glutamine/glycine/serine/tyrosine-rich region, *WT* wild-type, *Z* zinc finger motif. **f** Total cell lysates were treated with RNase (+) and immunoprecipitated (IP) with IgG (control) or anti-Fus antibodies. Samples were analyzed with the indicated antibodies. **g** Recombinant FMRP and FUS proteins were mixed and RNA was added in increasing concentrations. Protein mixtures were incubated and immunoprecipitated with anti-FUS antibody and subjected to immunoblotting with the indicated antibodies. **h** Schematic summary illustrating how RNA modulates FUS-FMRP binding. FUS and FMRP bind to each other and are both able to bind RNA (*left panel*). The interaction between FUS and FMRP decreases with increasing RNA concentrations (*middle panel*). Vice versa, degradation of RNA enhances the interaction between FUS and FMRP (*right panel*). *IB* immunoblot, *IC* input control

First, we used co-immunoprecipitation to confirm that Fus and Fmrp interact in motor neuron-like NSC-34 cells and primary cortical neurons (Fig. 6a, b). FUS and FMRP associate with many RNAs and other RNA-binding proteins. Thus, their interaction may be indirect and mediated by other proteins or RNAs. However, immunoprecipitation of recombinant FUS and FMRP proteins revealed a direct interaction in the absence of any other proteins, RNA molecules or cellular components (Fig. 6c). This indicates that FUS and FMRP can interact directly. To determine which region(s) of FUS mediate the interaction with FMRP, a series of FUS deletion mutants was generated and used in co-immunoprecipitation experiments. Full-length FUS and truncation mutants lacking the N-terminal QGSY region (Δ1–165) or the glycine-rich region (Δ165–276) bound FMRP. In contrast, FUS mutants lacking the C-terminal RGG-rich domain (Δ285–372) or the RNA recognition motifs (Δ360–501) did not interact with FMRP (Fig. 6d, e). Both FUS and FMRP are RNA-binding proteins and bind to and regulate many different RNAs [15, 32, 49]. We therefore investigated whether RNA modulates binding of FMRP to FUS. Interestingly, RNase treatment induced an increased interaction between Fus and Fmrp (441 % of RNase negative condition, *p* = 0.039) (Fig. 6f). To examine this effect of RNA in more detail, purified FUS and FMRP proteins were co-immunoprecipitated in the presence of an increasing amount of RNA. In line with the results from the RNase treatment, RNA application inhibited FUS-FMRP binding in a concentration-dependent manner (0.1 ng/μl = 70.4 %, *p* = 0.27; 0.5 ng/μl = 56.5 %, *p* = 0.08; 2.5 ng/μl = 23.3 %, *p* = 0.007; 12.5 ng/μl = 11.6 %, *p* = 0.003 and 62.5 ng/μl = 4.6 %, *p* = 0.002 of FUS-FMRP binding at 0 ng/μl RNA) (Fig. 6g). Together, these findings identify endogenous and direct binding of FUS and FMRP. Further, they suggest that this interaction can be modulated by RNA (Fig. 6h).

### Exogenous FMRP rescues neuromuscular junction and locomotor defects induced by ALS mutant FUS

**Fig. 7:**
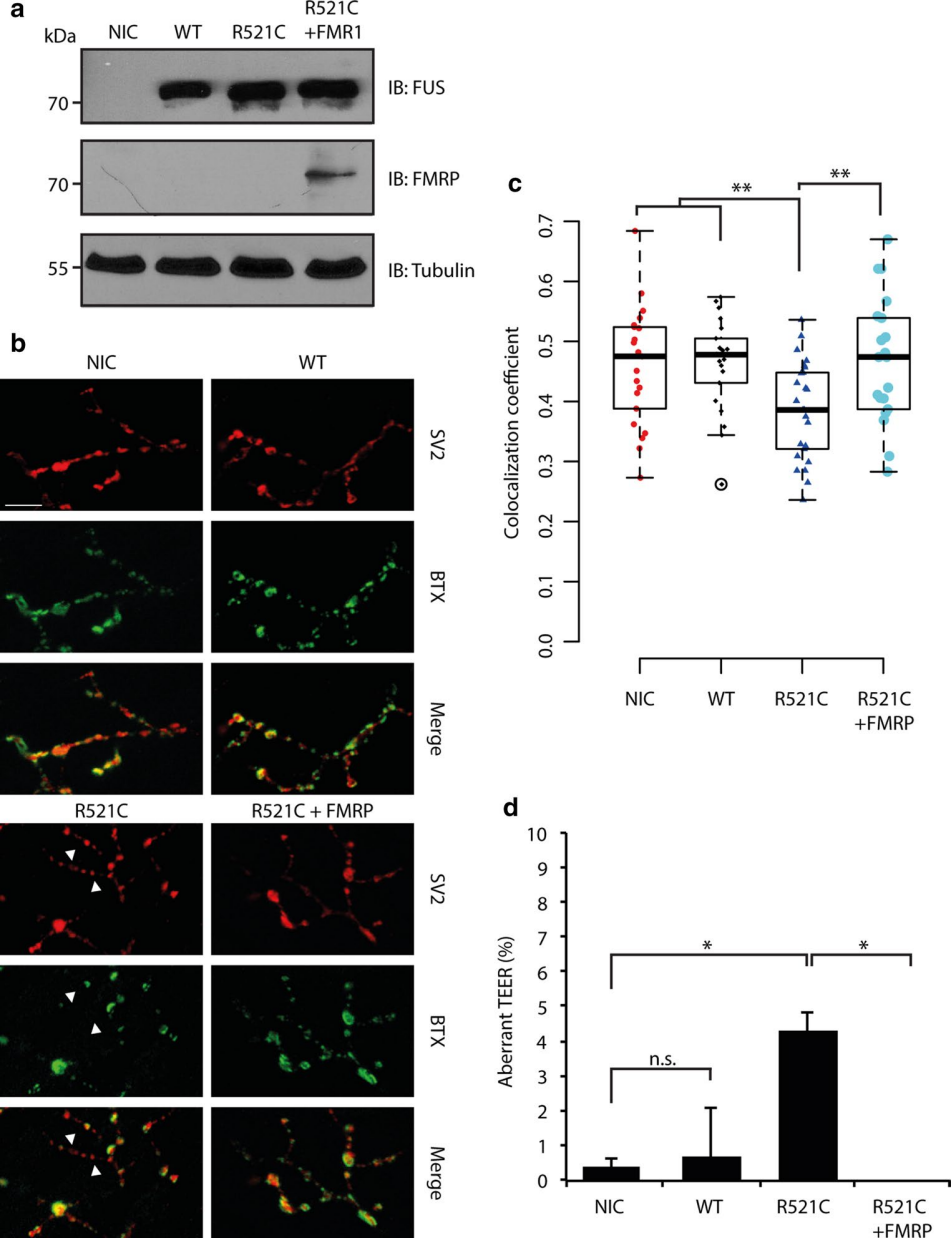
Exogenous FMRP rescues NMJ and locomotion defects caused by mutant FUS. **a** Western blot analysis of 72 hpf zebrafish embryo lysates using antibodies against human FUS or FMRP following injection of FUS WT, FUS R521C and/or FMR1 RNAs. Tubulin is used as a loading control. *NIC* non-injected control. **b** Representative images of neuromuscular junctions (NMJ) in 72 hpf NIC zebrafish embryos or following injection of FUS WT, FUS R521C, and FUS R521C and FMR1 RNAs. Anti-synaptic vesicles 2 (SV2) and anti-bungarotoxin (BTX) were used to label the pre- and postsynaptic compartments. *Arrowheads* indicate absence of colocalization between SV2 and BTX staining. **c** Quantification of the Pearson’s correlation coefficient for overlap between immunolabeling for pre- and post-synaptic markers, as shown in **b**. *n* = 20 per condition. *IB* immunoblot. **d** Quantification of touch-evoked escape responses (TEER) in zebrafish embryos at 72 hpf following injection of the indicated RNAs. Data is obtained over five experiments with *n* = 267 for NIC, *n* = 182 for FUS WT, *n* = 163 for FUS R521C and *n* = 111 for FUS R521C + FMR1. *p < 0.05, **p < 0.01, Fisher’s exact test. *Scale bar* 20 μm

Mutant FUS expression causes defects in synaptic transmission at the NMJ in zebrafish [1]. Similarly, loss of the *Drosophila* homolog of FMRP, dFXR, leads to NMJ defects in *Drosophila*, possibly through upregulation of the MAP1B homolog Futsch [95]. Interestingly, recent work identifies both Futsch and FMRP as modifiers of TDP-43-dependent toxicity in *Drosophila* [13, 14]. Here, we investigated whether FMRP is also able to modulate mutant FUS-induced ALS phenotypes in vivo, using zebrafish embryos as a model. First, we further characterized NMJ defects induced by mutant FUS to extend previous electrophysiological studies revealing abnormal synaptic transmission. Injected embryos were immunostained at 72 hpf with anti-synaptic vesicles 2 (SV2) antibodies, to stain the presynaptic compartment of NMJs, and alpha-bungarotoxin (BTX), to detect the postsynaptic compartment (Fig. 7a, b). As expected, in NIC and FUS WT injected embryos, considerable overlap was observed between pre- and postsynaptic staining (NIC: *R*^2^ = 0.46, FUS WT: *R*^2^ = 0.46, *p* = 0.91) (Fig. 7b, c). However, injection of FUS R521C caused a significant decrease in the overlap between pre- and postsynaptic markers (FUS R521C: *R*^2^ = 0.39, *p* < 0.01), hinting at a loss of NMJ integrity. We then asked whether co-expression of FMRP and FUS R521C would restore abnormalities in NMJ morphology. Indeed, co-expression of FMRP with FUS R521C restored NMJ defects induced by FUS R521C (Fig. 7a–c). Overlap of pre- and postsynaptic markers was similar to NIC and FUS WT, indicating normal NMJ integrity (FUS R521C + FMR1: *R*^2^ = 0.46, *p* = 0.86) (Fig. 7c).

To investigate the functional consequence of diminished NMJ integrity, we assessed zebrafish locomotion at 72 hpf (Fig. 7d). Developing zebrafish normally respond to touch by swimming away from the touch stimulus (known as the touch-evoked escape response, TEER). A low number of NIC or FUS WT larvae showed abnormal TEER (NIC: 0.37 %, FUS WT: 0.55 %, *p* = 0.99). In contrast, FUS R521C injection led to a significantly higher number of larvae with abnormal TEER (FUS R521C: 3.68 %, *p* < 0.05). In line with the ability of exogenous FMRP to restore NMJ integrity, zebrafish larvae co-injected with FUS R521C and FMRP displayed normal TEER. These data show that FMRP overexpression rescues mutant FUS-induced ALS phenotypes.

### FUS R521C induces increased synaptic expression of the FMRP target MAP1B

**Fig. 8:**
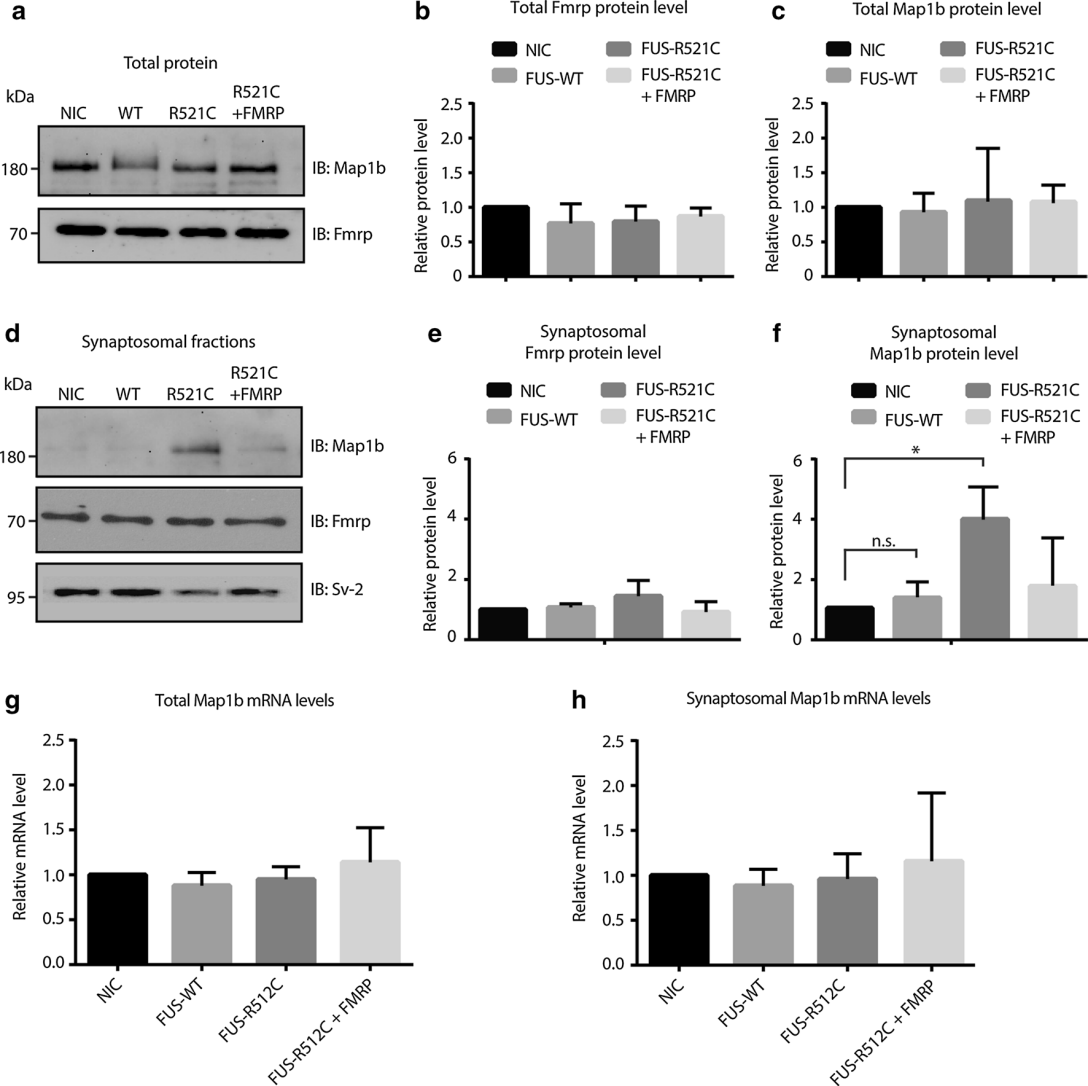
Synaptosomal expression of Map1b is increased by mutant FUS in vivo. **a** Total lysates were generated from 150 to 300 72 hpf zebrafish embryos injected with the indicated RNAs and subjected to immunoblotting with antibodies detecting endogenous Map1b and FMRP expression. Exogenously expressed human FMRP is not visible in this blot. *NIC* non-injected control, *WT* wild-type. **b**, **c** Quantification of band intensities as in **a** from at least 3 independent experiments. **d** Synaptosomal fractions were generated from 150 to 300 72 hpf zebrafish embryos injected with the indicated RNAs and subjected to immunoblotting with antibodies detecting endogenous Map1b, FMRP and Sv-2 expression. Exogenously expressed human FMRP is not visible in this blot. **e**, **f** Quantification of band intensities as in **d** from at least three independent experiments. *p < 0.05, n.s. = non significant, one-way ANOVA. **g**, **h** Quantitative RT-PCR for *Map1b* on total (**g**) or synaptosomal (**h**) RNA generated from 72 hpf zebrafish embryos injected with the indicated RNAs. Data are represented as mean ± SEM. *IB* immunoblot

Our previous work demonstrated that sequestration of SMN in mutant FUS aggregates causes an axonal depletion of SMN and defects in axonal morphology [28]. This study indicates that FMRP is also captured in mutant FUS aggregates. Therefore, we assessed whether axonal FMRP levels were decreased in primary motor neurons transfected with FUS R521C (as shown for Smn). However, quantification of the number of endogenous FMRP granules did not reveal differences between neurons transfected with GFP, FUS WT or FUS R521C (Fig. S7). As the subcellular distribution of FMRP appeared unaltered by mutant FUS, we next examined whether FMRP’s function as a regulator of local translation at synaptic sites was affected. Disruption of the repressive effect of FMRP on local translation could lead to increased translation of FMRP target mRNAs. To study this model, we used zebrafish embryos and focused on a well-characterized FMRP target, MAP1B. Consistent with our observation in transfected primary motor neurons, total FMRP protein levels were unchanged in zebrafish embryos expressing FUS R521C (Fig. 8a, b). Similarly, total expression of Map1b was unaffected (Fig. 8c). However, because FMRP regulates local translation at synaptic sites, we next examined Map1b protein levels in the synaptosomal compartment. Synaptosomes were isolated from injected zebrafish and analyzed by Western blot (Fig. S8). This analysis revealed a marked increase in synaptic Map1b levels following mutant FUS expression (Fig. 8d–f). Importantly, co-injection of FMRP RNA normalized enhanced Map1b levels induced by mutant FUS (Fig. 8d–f). Q-PCR analysis of total and synaptosomal fractions did not reveal changes in the mRNA expression of Map1b (Fig. 8g, h). Together, these data suggest that mutant FUS may impair translational repression of FMRP at the synapse, thereby increasing protein levels of FMRP targets such as MAP1B.

## Discussion

Our knowledge of the genetic etiology of ALS has increased significantly over the past years. However, our understanding of the molecular and cellular mechanisms underlying this disease is still incomplete. To further dissect the pathogenic mechanisms of ALS, we combined protein pull down and mass spectrometry approaches to define the interactomes of six ALS-associated proteins (WT and mutant forms). Experiments for different bait proteins were carried out in parallel and in triplicate resulting in the specific identification of several known and many novel interactors. Our data suggest that several different ALS-associated proteins act through common cellular pathways and that the ALS-associated mutations selected by us only affect interactome composition for OPTN and to a lesser extent for UBQLN2. In addition, our findings implicate FMRP, one of the common interactors of ATXN2, FUS, and TDP-43, in ALS pathogenesis caused by FUS mutations.

### Common pathways downstream of ALS-associated proteins

#### ATXN2, FUS and TDP-43

Our study shows that the interactomes of ATXN2, FUS and TDP-43 and those of OPTN and UBQLN2 share many binding partners. This suggests that these ALS-associated proteins contribute to common protein complexes and could regulate motor neuron viability or function via the same or similar cellular mechanisms. Furthermore, shared interactors of ATXN2, FUS and TDP-43 included other ALS-associated proteins such as Matrin3, hnRNPA1 and hnRNPA2B1, supporting the idea that many different ALS-associated proteins may cause disease via a common mechanism. The observation that ALS-associated mutations did not significantly alter the composition of ATXN2, FUS and TDP-43 interactomes indicates that the pathogenic effects of these select mutations may not be mediated by a complete loss of protein–protein interactions. However, it is plausible that mutations induce subtle but detrimental changes in protein–protein interactions that are not detected in our experimental setup. Our previous work and that of others demonstrates effects of ALS-associated mutations on protein–protein interactions [8, 26, 28, 52, 76, 88, 93]. Nevertheless, it is known that mutations also have other effects such as on subcellular localization (e.g., mutations in FUS) or aggregation propensity (e.g., mutations in TDP-43) of proteins [36]. Protein mislocalization or aggregation can lead to sequestration of binding partners resulting in their unavailability at their normal cellular site of action. In further studies, it will therefore be important to determine which of the binding partners identified here aberrantly localizes to ATXN2-, FUS- or TDP43-positive aggregates, such as shown in this study for FMRP, HuD, UPF1 and CAPRIN1.

Interacting proteins that were shared by ATXN2, FUS and TDP-43 included proteins involved in stress granule formation, as well as components of the spliceosome, initiation factors, ribosomal subunits and hnRNPs. In addition to shared interactors, each bait also bound a set of specific proteins. For example, FUS, but not ATXN2 or TDP-43, bound a number of proteins involved in DNA repair. This is in line with recent data implicating FUS in this process [54, 72, 84]. Our data confirm and extend these findings by revealing that FUS may be part of a DNA repair complex consisting of Histone H2AX (H2afx), DNA ligase 3 (Lig3), Parp1, Poly (ADP-ribose) polymerase 2 (Parp2), DNA polymerase beta (Polb), DNA repair protein XRCC1 (Xrcc1), X-ray repair cross-complementing protein 5 (Xrcc5), and X-ray repair cross-complementing protein 6 (Xrcc6). From a therapeutic perspective, proteins that bind several different ALS-associated proteins are interesting as they may provide a means for developing treatments aimed at larger groups of patients. However, interactors only binding a specific ALS protein are still valuable as they can help to provide insight into how ALS pathogenesis develops in specific subsets of patients and could be a starting point for the development of personalized medicine approaches.

#### OPTN and UBQLN2

In line with previous work, the OPTN interactome was composed of proteins playing a dual role in autophagy and NF-κB signaling. Interactors detected for UBQLN2 corresponded well with its known role in the ubiquitin-proteasome system [45] and supported its suggested roles in ERAD [88], endosomal trafficking [61, 64], NF-κB signaling and autophagy [64, 66, 85]. Significant overlap was detected between the interactomes of OPTN and UBQLN2. In line with previous work localizing both proteins to endosomal and autophagic vesicles [64], both OPTN and UBQLN2 bound Rab1, Rab6 and Tbc1d15. However, the most striking overlap between the OPTN and UBQLN2 interactomes included regulators of autophagy including Sqstm1, Tax1bp1, Tbk1 and Vcp, which also play a role in NF-κB signaling. Some of these interactions were reported previously (OPTN-SQSTM1 [85], OPTN-TBK1 [59], TBK1-SQSTM1 [67], VCP-ubiquilins [51], but our study is the first to show that all these proteins are part of a common complex. The observation that Sqstm1, Tbk1 and Vcp are shared interactors of OPTN and UBQLN2 is intriguing as mutations in these genes have been associated with ALS [9, 22, 25, 37]. Future analyses of the interactomes of wild-type and mutant SQSTM1, TBK1 and VCP are needed to pinpoint how these proteins cooperate in a common complex with OPTN and UBQLN2 and how alterations in this complex may lead to ALS or other neurodegenerative diseases.

In contrast to ATXN2, FUS and TDP-43, mutations in OPTN and, to a lesser extent, UBQLN2 induced loss or strengthening of specific protein–protein interactions. We found that the E478G mutation in OPTN causes loss of binding to 13 interactors, while interactions with other proteins, e.g., Tbk1, 14-3-3 proteins or dynein, were unaffected. A number of different processes are disrupted by the E478G mutation in OPTN, including TNF-α-induced NF-κB signaling [53], Salmonella autophagy [85], autophagic clearance of damaged mitochondria [87], and endosomal vesicle formation [64]. Our data suggest that these defects may result from the inability of OPTN E478G to bind specific interactors. The location of the E478G mutation in the ubiquitin-binding domain of OPTN supports the idea that the interactions influenced by this mutation may be ubiquitin-dependent. Mutant UBQLN2 P497H showed increased affinity for Cltc. This supports previous work suggesting that the P497H mutation in UBQLN2 may disrupt receptor endocytosis via increased affinity for Cltc [61]. Endocytosis is essential for neuronal functioning [86] and deregulation of endosomal trafficking has been related to different neurological disorders including Huntington’s disease and Charcot–Marie–Tooth disease [10].

#### C9orf72

The function of C9orf72 is largely unknown, but previous work indicates that C9orf72 contains a DENN domain and may play a role in autophagy and endosomal trafficking by interacting with Rab proteins [21, 50, 92]. Co-immunoprecipitation from SH-SY5Y cells revealed putative interactions between C9orf72 and Rab1, Rab5, Rab7, Rab11, Ubql1, Ubqln2, hnRNPA1 and hnRNPA2B1 [21]. Our interactomics study, however, did not detect these interactors or other proteins involved in autophagy or endocytosis but rather unveiled enrichment for proteins with mitochondrial functions. One explanation for this apparent discrepancy in C9orf72 interactome composition is that it is strongly influenced by cell type or endogenous expression level. Although endogenous C9orf72 levels are generally low and our study used exogenous C9orf72 expression to detect interactors, we have performed biotin-streptavidin pull downs for over 40 protein baits but never detected a striking enrichment for mitochondrial proteins (e.g., [28, 78, 80]). The specificity of our approach is further confirmed by the presence of C9orf72 in mitochondrial fractions following subcellular fractionation and detection by recently reported C9orf72-specific antibodies ([46]; Fig. S2). It should be noted that many of the currently available C9orf72 antibodies are rather non-specific, including some of those used for identifying C9orf72 interactors (Fig. S9) [83]. Another recent study has reported interactions between two different GFP-tagged C9orf72 isoforms and importin-B1 or Ran-GTPase in N2A cells [89]. These interactors were also identified in our study, but did not pass our stringent selection criteria. Interestingly, non-coding *C9ORF72* repeat expansions may cause ALS by affecting nuclear transport through importin- and RAN-dependent mechanisms [24, 40, 94]. Although neural-specific *C9orf72* knockout mice do not display motor neuron degeneration or motor behavior defects [46], it is possible that loss-of-function mechanisms cooperate with gain-of-function defects caused by repeat expansions and that this cooperation causes motor neuron degeneration through dysregulation of importins and Ran-GTPases. Further work is needed to address these and other possible mechanisms and to dissect the precise subcellular distribution of C9orf72 and its link to for example mitochondria and endocytotic vesicles.

### FMRP is a common target in ALS pathogenesis

To show that interactomics analyses can lead to the identification of shared interactors of different ALS-associated proteins that are relevant for ALS pathogenesis, we focused on FMRP. FMRP is a translational repressor at synaptic sites [15] and FMRP overexpression rescues TDP-43 toxicity in *Drosophila* [14]. Our study identified FMRP as an interactor of TDP-43, ATXN2 and FUS. Therefore, to explore a more general role for FMRP in ALS pathogenesis we studied the potential link between FMRP and mutant FUS. We show endogenous, direct and RNA-sensitive interactions between FMRP and FUS, and found that FMRP localizes to mutant FUS-positive cytoplasmic aggregates in spinal motor neurons. FMRP has been previously shown to localize to mutant FUS granules in mouse fibroblast cells [91], but not in SH-SY5Y cells [74]. Further, exogenous expression of FMRP rescued defects in NMJ integrity and motor behavior in zebrafish embryos caused by mutant FUS.

How does FMRP modulate cellular defects caused by mutant FUS or TDP-43? Previous studies show that sequestration of SMN in cytosolic aggregates by mutant FUS causes its axonal depletion [28]. Other ALS-associated mutations have also been proposed to act by depleting proteins from their site of action in the cell [30, 58]. However, mutant FUS did not cause reduced FMRP levels in mouse motor neuron axons or synaptosomes derived from zebrafish embryos. Nevertheless, protein but not mRNA expression of a well-characterized FMRP target, Map1b, was increased at the synapse. MAP1B is a microtubule stabilizing protein regulating NMJ morphology [71]. Overexpression of the *Drosophila* MAP1B homolog Futsch induces NMJ abnormalities and impaired neurotransmission [95]. Intriguingly, overexpression of wild-type or mutant TDP-43 causes a small synaptic downregulation of Futsch in *Drosophila*, which can be reversed by FMRP overexpression. These data together with our results suggests that strict control of synaptic MAP1B expression is essential for maintaining motor neuron morphology and survival. Although it is unclear why FUS and TDP-43 have opposing effects on local translation of MAP1B, it is possible that both proteins affect MAP1B via distinct mechanisms. For example, although TDP-43 is present on polysomes [13], we only detected weak to no FUS expression in polysomal fractions (Fig. S10). But how do FUS mutations affect FMRP and its downstream mRNA targets? One possibility is that the increase in cytosolic FUS caused by ALS mutations leads to enhanced binding between FUS and FMRP, thereby preventing FMRP from binding to its target mRNAs on stalled polyribosomes leading to increased translation of MAP1B [15]. In line with this model, preliminary studies reveal enhanced binding between mutant FUS and FMRP and show increased expression of mutant as compared to wild-type FUS in FMRP granules in primary motor neurons (Fig. S11; A. M. B., M. K. and R. J. P., unpublished observations). Alternatively, FUS may alter the localization of FMRP at the synapse or by itself bind and control translation of *MAP1B* mRNA. Future studies are required to further examine the role and mechanism-of-action of FMRP in ALS pathogenesis.

In conclusion, we report a novel dataset comprised interactomes of six ALS-associated proteins that will provide a framework for future studies into the pathogenic mechanisms underlying ALS or other diseases linked to the proteins studied here [e.g., frontotemporal dementia (FTD; C9orf72, FUS, TDP-43) or SCA2 (ATXN2)]). We show that the interactors of multiple different ALS-associated proteins converge on a limited number of molecular complexes and related processes, namely RNA metabolism (ATXN2, FUS, hnRNPA1, hnRNPA2B1, MATR3 and TDP-43), autophagy and NF-κB signaling (OPTN, SQSTM1, TBK1, UBQLN2 and VCP) and mitochondrial functioning (C9orf72). Finally, the observation that FMRP, a shared interactor of ATXN2, FUS, and TDP-43, rescues FUS toxicity in an in vivo ALS model demonstrates that comparative interactomics analyses can aid in the identification and characterization of disease relevant proteins. This work showing that FMRP can reverse pathogenic effects caused by mutant FUS and TDP-43 identifies FMRP as an interesting therapeutic target in ALS.

## Electronic supplementary material

Below is the link to the electronic supplementary material.

Supplementary material 1 (PDF 12526 kb)

Supplementary material 2 (PDF 215 kb)
