# Supplementary material for: Comparative interactomics analysis of different ALS-associated proteins identifies converging molecular pathways
Source: Acta Neuropathol. 2016 May 10;132:175–96. doi: 10.1007/s00401-016-1575-8 (PMC4947123; doi:10.1007/s00401-016-1575-8)
Supplement: Supplementary file 1 — Supplementary material 1 (PDF 12526 kb) [file 401_2016_1575_MOESM1_ESM.pdf]

Supplementary Material

Comparative interactomics analysis of different ALS-associated proteins identifies converging molecular pathways

Anna M. Blokhuis, Max Koppers, Ewout J.N. Groen, Dianne M.A. van den Heuvel, Stefano Dini Modigliani, Jasper J. Anink, Katsumi Fumoto, Femke van Diggelen, Anne Snelting, Peter Sodaar, Bert M. Verheijen, Jeroen A.A. Demmers, Jan H. Veldink, Eleonora Aronica, Irene Bozzoni, Jeroen den Hertog, Leonard H. van den Berg and R. Jeroen Pasterkamp

Corresponding author:

R. J. Pasterkamp

Department of Translational Neuroscience, Brain Center Rudolf Magnus, University Medical Center Utrecht, Utrecht, The Netherlands

r.j.pasterkamp@umcutrecht.nl

Includes:

Supplementary Figures S1-S11

Supplementary Tables S3 and S4

Extended materials and methods

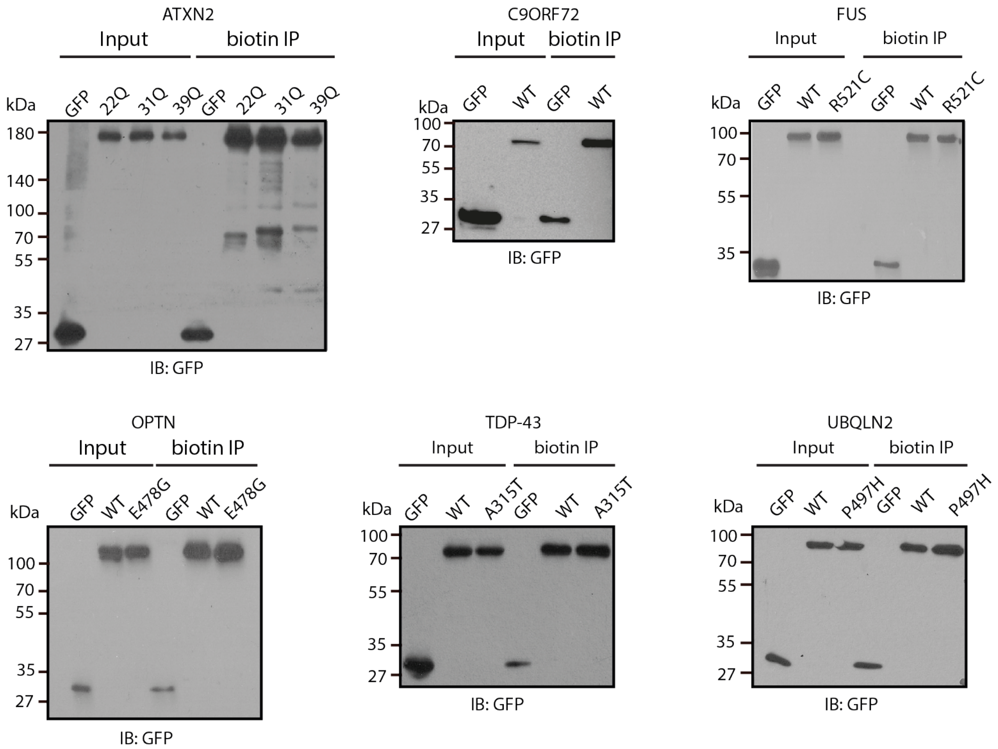

Fig. S1 Western blot analysis of input and immunoprecipitation (IP) samples following biotin IPs of N2A cells transfected with the indicated constructs and BirA. IB, immunoblot.

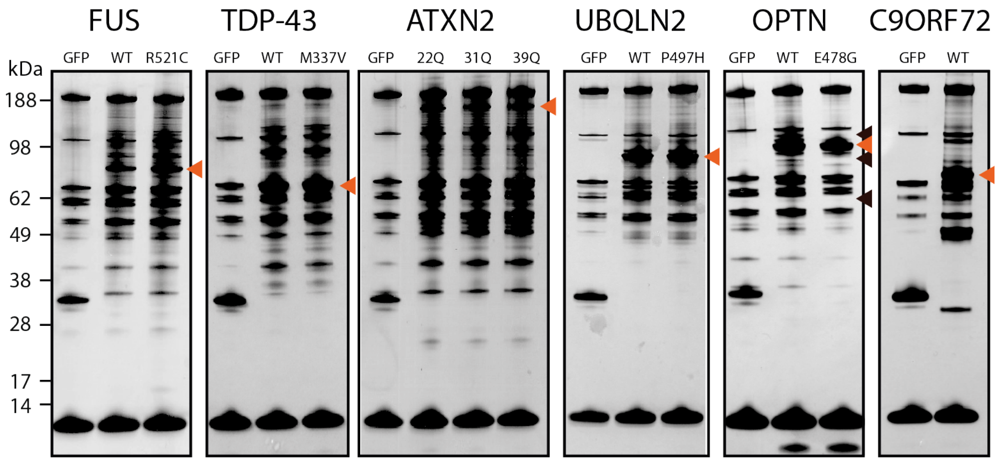

Fig. S2 Protein complexes purified by bioIP were separated using gel electrophoresis and proteins were visualized using silver staining. Orange arrows indicate bait proteins, black arrows point to interactors lost in OPTN E478G compared to OPTN wild-type (WT).

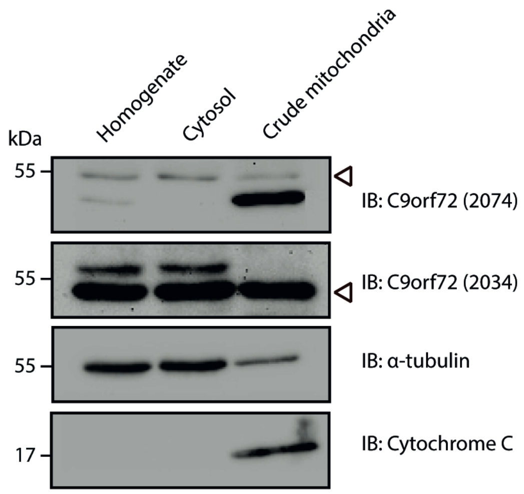

Fig. S3 Western blot analysis of subcellular fractions isolated from Neuro2A cells. Equal amounts of protein from each fraction were loaded and immunoblotted with anti-C9orf72 (2074 and 2034 [1]), anti-α-tubulin (cytoplasmic marker) or anti-cytochrome C (mitochondrial marker) antibodies, as indicated. Endogenous C9orf72 (white arrowheads) is present in the crude mitochondrial fraction.

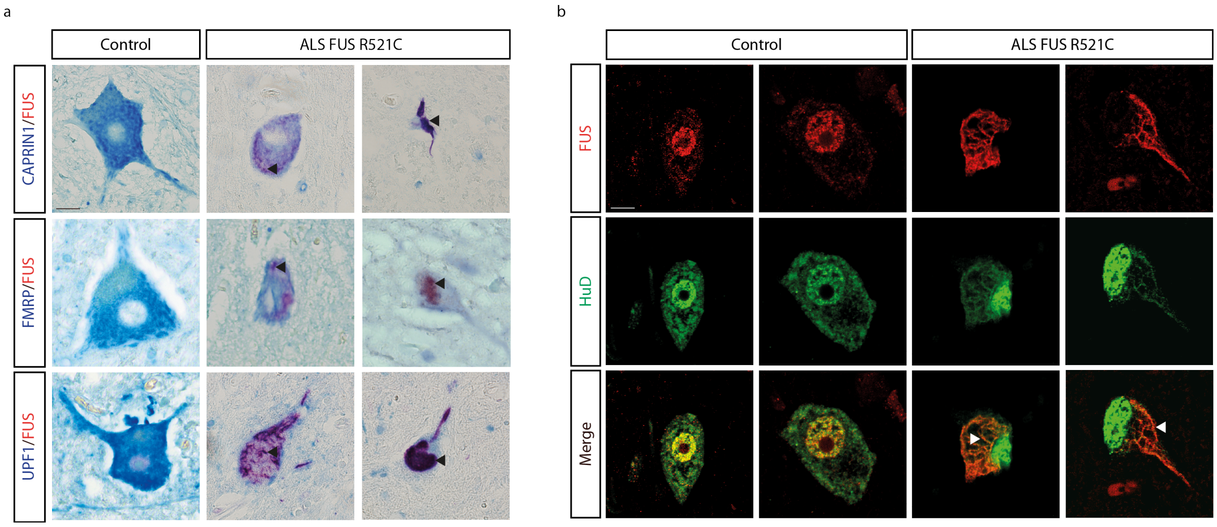

Fig. S4 Immunohistochemistry (**a**) or immunofluorescence (**b**) showing spinal cord motor neurons of ALS patients harboring FUS R521C mutations and controls. Sections were stained with anti-FUS antibody (**a**, **b**; red) and antibodies against CAPRIN1, FMRP, UPF1 (**a**; blue) or HuD (**b**; green), as indicated. Arrowheads indicate colocalization of CAPRIN1, FMRP, UPF1 (black arrowheads) and HuD (white arrowheads) with FUS aggregates in ALS patients. Scale bar, 20 µm.

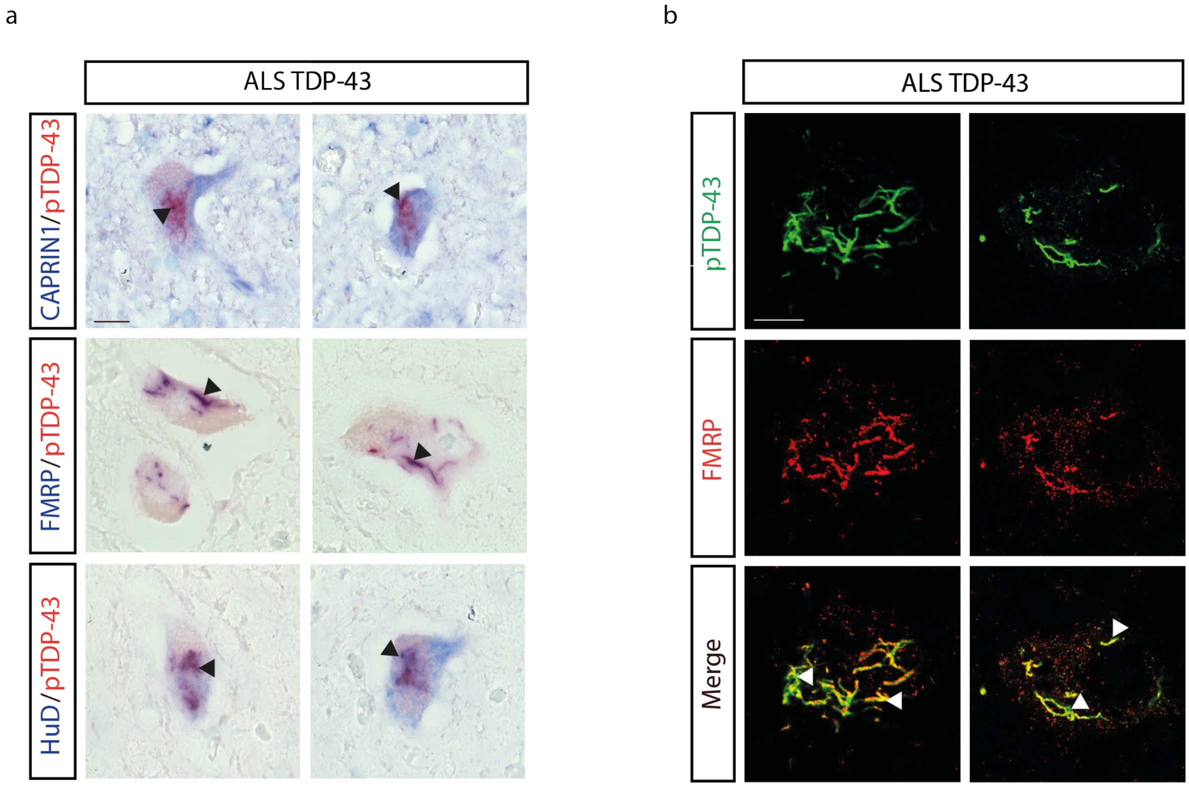

Fig. S5 Immunohistochemistry (**a**) or immunofluorescence (**b**) showing spinal cord motor neurons of ALS patients harboring TDP-43 inclusions. Sections were stained with anti-pTDP-43 antibody (**a**; red, **b**; green) and antibodies against CAPRIN1, FMRP, HuD (**a**; blue) or FMRP (**b**; red), as indicated. Arrowheads indicate colocalization of CAPRIN1, FMRP, UPF1 (black arrowheads) and FMRP (white arrowheads) with TDP-43 aggregates in ALS patients. Scale bar, 20 µm.

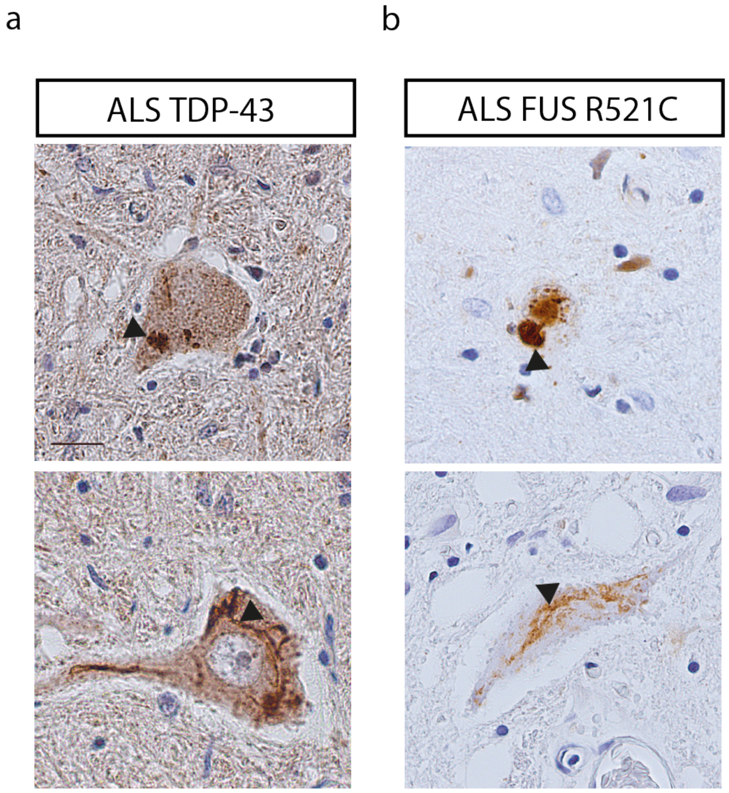

Fig. S6 Immunohistochemistry showing spinal cord motor neurons of ALS patients harboring TDP-43 (**a**) and FUS inclusions, as used in this study (**b**). Upper panels show compact inclusions and lower panels show thread-like inclusions. Sections were stained with anti-pTDP-43 antibody (**a**) or anti-FUS antibody (**b**). Scale bar, 20 μm.

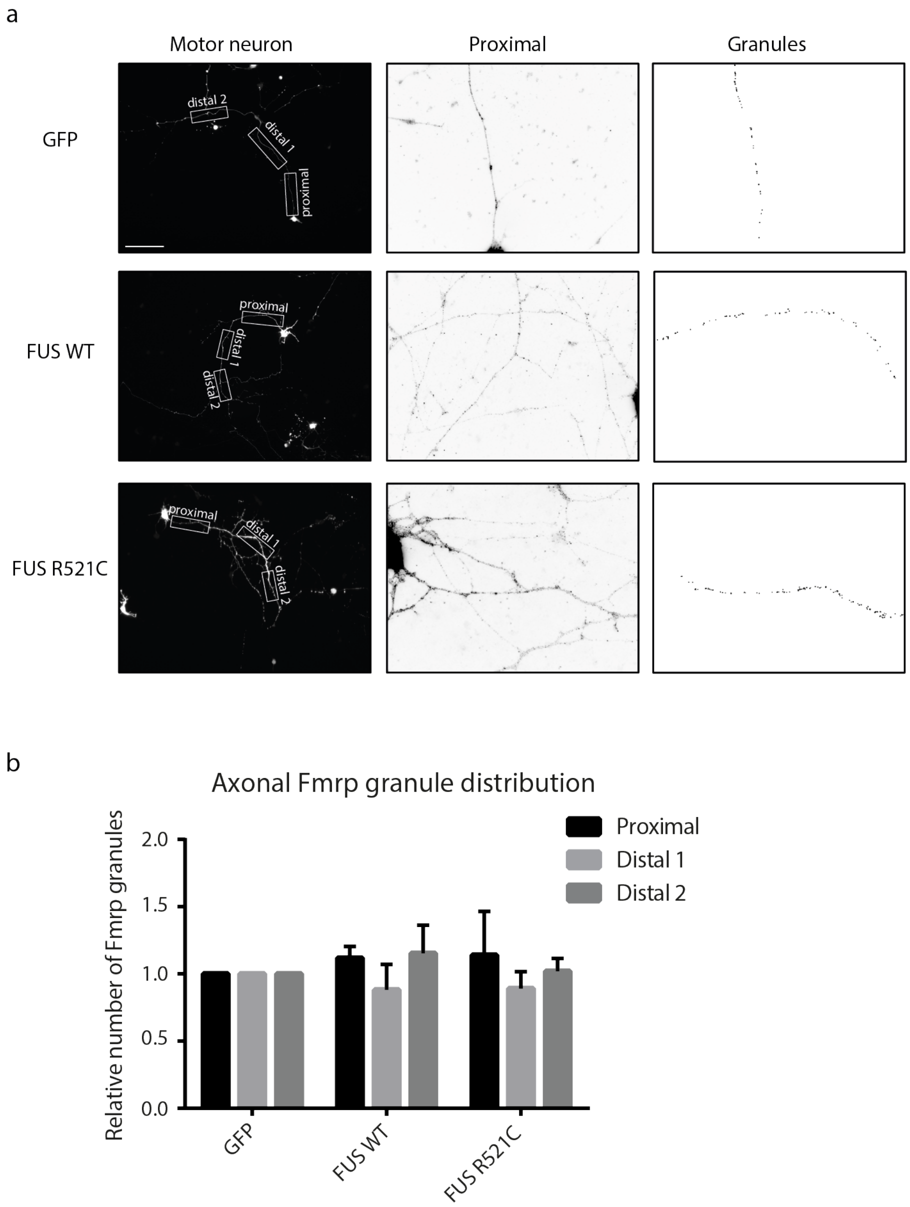

Fig. S7 Distribution of axonal FMRP granules is not affected by mutant FUS. (**a**) Dissociated primary motor neurons were generated from E13 mouse embryos and transfected with GFP (control), FUS WT or FUS R521C constructs at 2 days *in vitro*. Representative images are shown. A proximal segment and two distal segments were selected in each axon and high magnification images were acquired. Middle panels represent inverted images of the proximal part of the axon. Right panels represent processed images using the 'analyze particles' function in ImageJ. (**b**) Relative quantification of the number of axonal FMRP granules at different parts of the motor neuron axon shows no statistically significant differences between different groups (one-way ANOVA). Granules were counted in > 10 neurons per condition derived from 3 independent experiments. Data are shown as means ± S.E.M.. Scale bar, 60 µm.

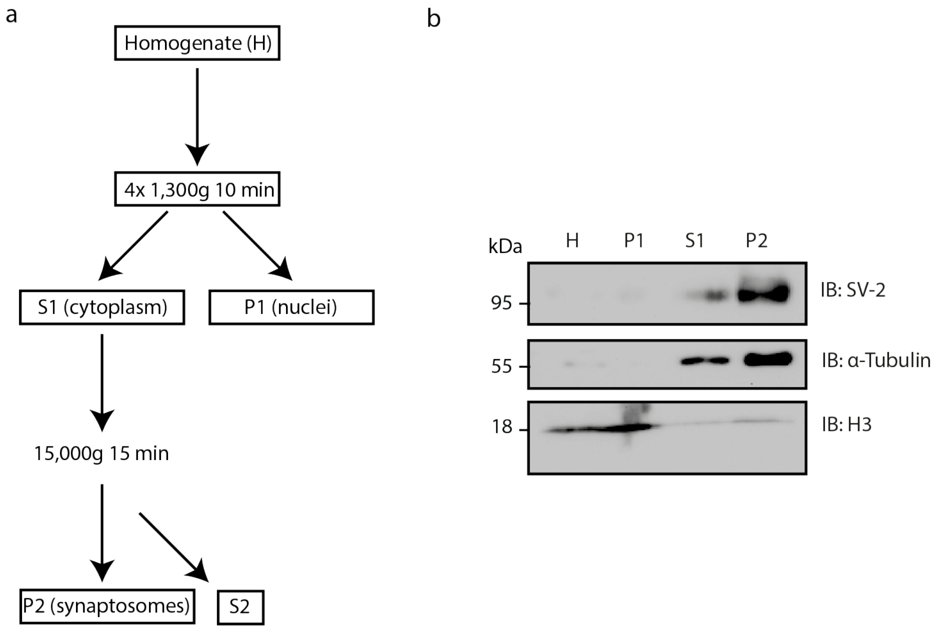

Fig. S8 Western blot analysis of subcellular fractions isolated from 72 hpf zebrafish embryos. (**a**) Schematic overview of synaptosomal isolation. (**b**) Equal amounts of protein were loaded and immunoblotted with anti-synaptic vesicles-2 (SV2; synaptosomal marker), anti-α-tubulin (cytoplasmic marker) or anti-histone H3 (nuclear marker) antibodies, as indicated. The synaptosomal fraction is enriched for SV2 and the nuclear fraction is enriched for histone H3. H, homogenate/total protein fraction; S1, cytoplasmic fraction; P1, nuclear fraction; P2, synaptosomal fraction.

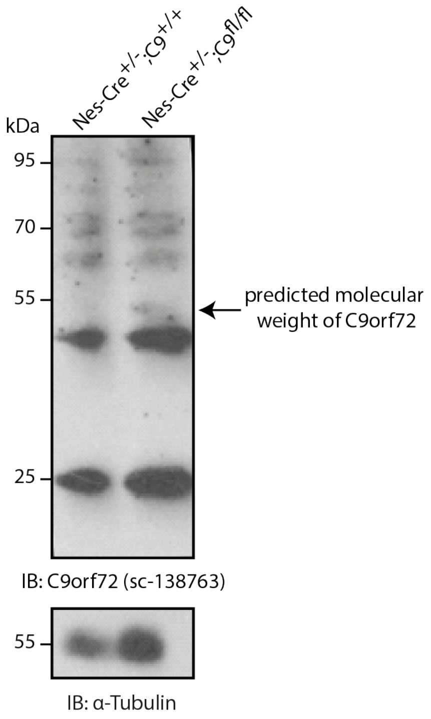

Fig. S9 Western blot analysis of whole brain lysates from Nestin-cre$^{+/-}$;C9orf72$^{+/+}$ and Nestin-cre$^{+/-}$;C9orf72$^{fl/fl}$ mice [1]. Whole brain lysates were immunoblotted with anti-C9orf72 (sc-138763, Santa Cruz) and anti-α-tubulin was used as a loading control. Banding pattern are similar in Nestin-cre$^{+/-}$;C9orf72$^{+/+}$ (wild-type) and Nestin-cre$^{+/-}$;C9orf72$^{fl/fl}$ (neural-specific knockout) mice.

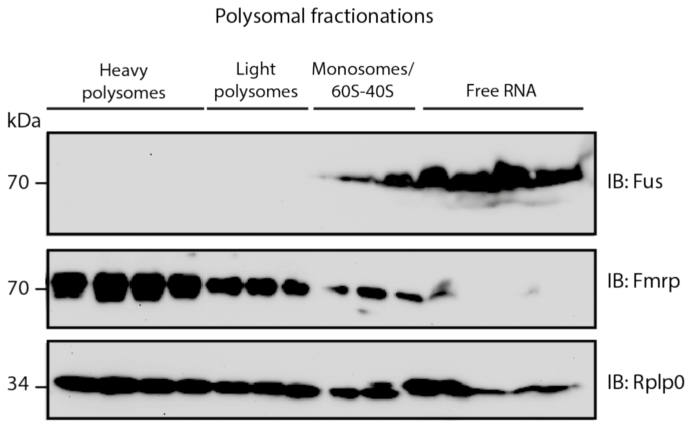

Fig. S10 Western blot analysis of polysomal fractionations from Neuro2A cells treated with cyclohexamide. Samples from different polysomal fractions were immunoblotted with anti-FUS, anti-FMRP, or anti-Rplp0 (ribosomal marker) antibodies. As expected, Fmrp is mainly present in the heavy and light polysomal fractions. Fus is largely absent from these fractions.

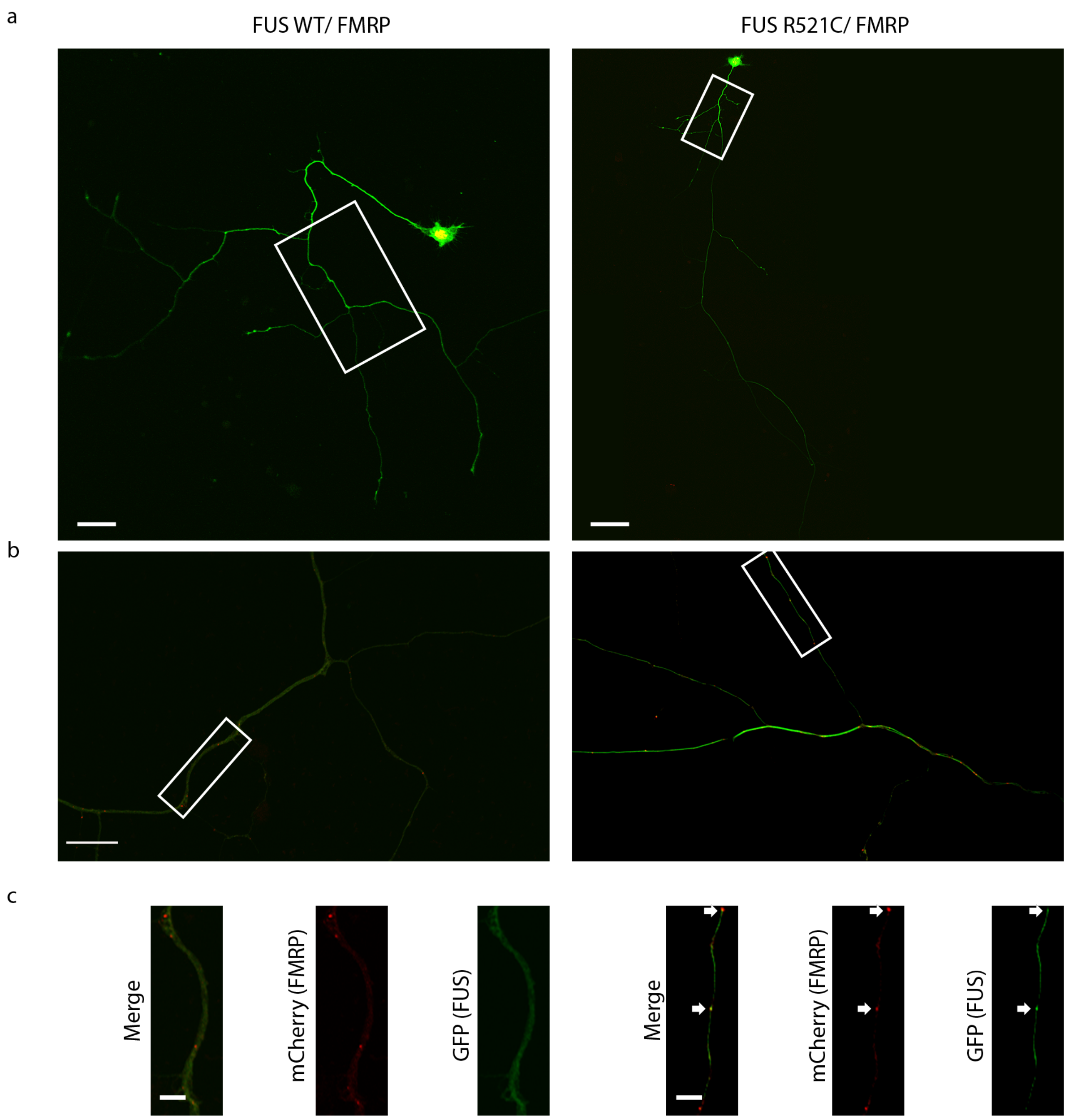

Fig. S11. Increased colocalization of mutant FUS with FMRP in granules. (**a)** Dissociated primary motor neuron cultures generated from E13.5 mouse embryos were co-transfected with DNA constructs expressing mCherry-tagged FMRP and GFP-tagged FUS wild-type (FUS WT, left panel) or FUS R521C (FUS R521C, right panel) at 5 days *in vitro* (DIV5) and fixed at DIV7. Scale bar, 50 µm (left panel), 100 µm (right panel). (**b)** High magnification of neurons as indicated by the white rectangles in (**a**). Scale bar, 20 µm. (**c)** Detail of (**b**) as indicated by the white rectangles. For FUS WT, no GFP-FUS signals were detected in mCherry-FMRP-positive granules in mouse spinal motor neuron axons. In contrast, for FUS R521C, partial colocalization of GFP-FUS and mCherry-FMRP was observed in axonal FMRP granules (arrows). Scale bar, 5 µm.

Supplementary Table 3:

Characteristics of patients and controls used for immunohistochemistry of spinal cord sections.

Patient	Age at onset	Site of onset	Disease duration (months)	FUS mutation
1	39	spinal	~24*	R521C
2	39	bulbar	20	R521C
3	65	spinal**	33	-
4	40	spinal**	20	-
5	59	spinal**	27	-

*by approximation, **TDP-43 inclusions

Control	Age at death	Cause of death
1	66	Peritonitis/sepsis
2	67	Arrhytmia
3	65	Myocardial infarction

Supplementary Table 4:

Analysis of colocalization of mutant FUS and its interactors in aggregate-like structures.

Interactor	Coaggregation in COS	Coaggregation in pr MN	Coaggregation in ALS-FUS spinal cord
Fmrp	+	+	+
Upf1	+	+	+
Caprin1	+	+	+
HuD	+	+	+
Pabpc4	+	+	-
Dhx9	+	+	-

Overview of the presence (+)/absence (-) of colocalization of FUS R521C and Fmrp, Upf1, Caprin1, HuD, Pabpc4 or Dhx9 in aggregate-like structures in transfected COS cells (transfected with FUS R521C), transfected primary motor neurons (transfected with FUS R521C) and human spinal cord material (from ALS patients carrying FUS R521C mutations).

References

1. Koppers M, Blokhuis AM, Westeneng HJ et al (2015) C9orf72 ablation in mice does not cause motor neuron degeneration or motor deficits. Ann Neurol 78:426-438. doi 10.1002/ana.24453

Extended material and methods

Antibodies

Rabbit anti-FUS (A300-302A) and rabbit anti-PABPC4 (A301-466A) antibodies were from Bethyl Laboratories. Mouse monoclonal anti-Ataxin-2 (611378) antibody was from BD-Transduction. Rabbit anti-RHA (ab26271), rabbit anti-FMRP (ab17722), rabbit anti-OPTN (ab23666), rabbit anti-UPF1 (ab109363), chicken anti-GFP (ab13970), rabbit anti-H3 (ab1791) and rabbit anti-GFP (ab290) antibodies were from Abcam. Rabbit anti-Caprin1 (15112-1-AP) and rabbit anti-TDP-43 (10782-2-AP) antibodies were from ProteinTech. Mouse anti-HuD antibody (E-1) and mouse anti-FUS (sc47711) were from Santa Cruz. Mouse anti-FMRP (#MAB2160) was from Millipore. Mouse anti-UBQLN2 (H00029978-M03) and rabbit anti-FUS (NB100-2599) were from Novus Biologicals. Mouse anti-alpha-tubulin was from Sigma-Aldrich (T5168). Mouse anti-phospho TDP-43 (pS409/410; TIP-PTD-M01) was from Cosmo Bio. Rabbit anti-UPF1 antibody was a kind gift from Jens Lykke-Andersen (University of California San Diego, USA). Rabbit anti-MAP1B antibody 489-3 was a kind gift from Ithzak Fischer (Dexel University, USA). Rabbit anti-C9ORF72 antibodies C9-2034 and C9-2074 were generated as described by Koppers et al., (2015) [46]. Mouse anti-SV-2 was from the Developmental Studies Hybridoma Bank (DSHB), Iowa City, IA and rabbit anti-Rplp0 (213174) was obtained from US Biologicals.

Plasmids

Expression constructs were generated by cloning the human cDNAs of *FUS*, *TDP-43*, *ATXN2* 22Q, *ATXN2* 31Q, *ATXN2* 39Q, *OPTN*, *UBQLN2* and *C9orf72* into a modified eGFP vector containing a biotin sequence N-terminal to GFP [35]. *FUS*, *TDP-43*, *OPTN*, and *UBQLN2* cDNAs were obtained from Origene; *C9orf72* cDNA corresponding to the ‘long’ isoform (481aa) was obtained as described [46]; *ATXN2* 22Q, 31Q and 39Q cDNAs were a kind gift from Aaron Gitler (Stanford University, USA) and *BirA* cDNA was a kind gift from Casper Hoogenraad (Utrecht University, The Netherlands). The chicken beta-actin mCherry-FMRP construct was a kind gift from Rob Willemsen (Erasmus University Rotterdam, The Netherlands).

The QuickChange Site-directed Mutagenesis kit (Stratagene) was used to introduce ALS-associated mutations into FUS, TDP-43, OPTN and UBQLN2.

Cell culture and transfection

Neuro2A (N2A) cells (mouse neuroblastoma cells) were maintained in DMEM low-glucose medium (Gibco) supplemented with 10% heat-inactivated fetal calf serum (Lonza), 2 mM L-glutamine, and 1x penicillin/streptomycin. Human Embryonic Kidney (HEK) 293 and NSC-34 cells were maintained in DMEM high-glucose medium (Gibco) supplemented with 10% heat-inactivated fetal calf serum (Lonza), 2 mM L-glutamine, and 1x penicillin/streptomycin. Cells were transfected using X-tremeGENE HP (Roche), according to the manufacturer's instructions. Transfection efficiency was 80-90%.

Biotin-streptavidin pulldown

Immunoprecipitation of biotin-tagged proteins was performed as described previously [16, 28]. Paramagnetic streptavidin beads (Dynabeads M-280, Life Technologies) were pre-blocked in blocking buffer (20 mM Tris–HCl pH 8.0, 150 mM KCl, 20% glycerol, 200 ng/µl purified chicken serum albumin (Sigma-Aldrich)). N2A cells transfected to express the biotin ligase BirA and different biotin-tagged cDNA constructs were lysed in lysis buffer (20 mM Tris–HCl, pH 8.0, 150 mM KCl, 1% Triton X-100, 0.2 µg/µl CEA, phosphatase inhibitor cocktail (Sigma), complete protease inhibitor cocktail (Roche)), incubated 10 minutes on ice and centrifuged at 13,200 rpm for 10 minutes at 4°C. Supernatants were incubated with blocked paramagnetic streptavidin beads for 30 minutes at 4°C followed by 4 washes in washing buffer (20 mM Tris–HCl, pH 7.5, 150 mM KCl, 0.1% Triton X-100, proteinase inhibitor cocktail) and precipitated proteins were eluted by boiling in elution buffer (1x NuPage LDS sample buffer, 10 mM DTT) for 5 minutes at 90°C. It is important to note that cell lysis could allow bait proteins to interact with proteins that they normally would not be able to bind (e.g. because of their localization in distinct cellular compartments). While this is an important point to consider, so far we have performed bioIPs for >40 different bait proteins (including cytoplasmic, nuclear or membrane-associated proteins) and never found strong evidence for such post-lysis artifacts.

In-gel analysis

Samples were separated in a NuPAGE Novex 4–12% Bis/Tris gradient gel following the manufacturer's instructions. For silver staining, gels were fixed in 50% methanol for 30 minutes, followed by incubation in 10 µM DTT for 15 minutes and incubation in 0.1% (w/v) $AgNO_3$ for 20 minutes. Subsequent gels were developed

using 0.25 M anhydrous sodium carbonate containing 0.02% (w/v) formaldehyde. For mass-spectrometry analysis, gels were stained using GelCode blue stain reagent (Pierce).

LC-MS/MS analysis

Mass-spectrometry analysis of interacting proteins was done as described previously [96]. Raw data were analyzed by MaxQuant (version 1.4.1.2) [12] using default settings with the additional option label free quantitation ('LFQ') selected. The generated 'proteingroups.txt' table was filtered for contaminants and reverse hits. For interactor identification, t-test-based statistics was applied on the LFQ data using Perseus in the MaxQuant software suite. First, the logarithm (log 2) of the LFQ values were taken, resulting in Gaussian distributions of the data. This allowed imputation of missing values by normal distribution (width=0.3, shift=1.8), assuming these proteins were close to the detection limit. Statistical outliers for the ATXN2, C9orf72, FUS, OPTN, TDP-43 or UBQLN2 groups compared to the control group were then determined using a two-tailed Welch's t-test. Multiple testing correction was applied by using a permutation-based false discovery rate (FDR) method. GO analysis was performed using PANTHER [57]. These outliers were then defined as specific interactors for the bait protein in question, since their LFQ values were statistically different from the corresponding values in the control groups and identified by at least two unique tryptic peptides. Another requirement for categorizing proteins as *bona fide* interactors was that these protein hits should be present in all individual triplicates.

Immunoprecipitation and immunoblotting

For the co-immunoprecipitation of endogenous protein complexes, N2A or NSC-34 cells from one 10-cm dish were lysed in 150 µl lysis buffer (20 mM Tris–HCl pH 7.5, 150 mM NaCl, 1% NP-40, 10% glycerol, complete protease inhibitor cocktail (Roche)), incubated on ice for 10 minutes and centrifuged at 13,200 rpm for 10 minutes at 4°C. Lysates were incubated for 1 h at 4°C using 1 µg antibodies. Samples were incubated with protein A or protein G magnetic beads for 30 minutes followed by 4 washes in lysis buffer. Precipitated proteins were eluted by boiling in NuPage LDS sample buffer containing 10 mM DTT for 5 minutes at 90°C. For RNA dependency experiments, lysates were first treated with RNAse A (Roche) for 30 minutes at 4°C. Recombinant FUS (Origene) and FMRP (Abnova) proteins were purchased to study direct protein-protein interactions. Proteins were mixed in buffer (10% glycerol, 0.5% NP40, 100 mM glycine, 25 mM Tris-HCl, pH 7.4) and incubated for 1 h with protein A agarose beads pre-incubated with rabbit anti-FUS antibody.

Immunoprecipitation was performed as described above. For RNA dependency experiments, total RNA was isolated from HEK293 cells using the RNAeasy mini kit (Qiagen) according to the manufacturer's instructions and added to the protein mixture at concentrations ranging from 0.1 to 62.5 ng/µl. For the generation of crude mitochondrial fractions, N2A cells were homogenized with a potter in homogenization buffer (0.25 M sucrose, 0.2 mM EDTA, 20 mM HEPES pH 7.4, complete protease inhibitor cocktail (Roche)) and centrifuged twice at 700 g for 10 minutes at 4°C. Supernatant was then centrifuged at 7,000 g for 10 minutes at 4°C after which the pellet was resuspended in homogenization buffer and centrifuged again at 7,000 g for 10 minutes at 4°C. The resulting pellet was resuspended again in homogenization buffer and centrifuged at 10,000 g for 10 minutes at 4°C to obtain the pellet containing crude mitochondria.

Proteins were separated in a SDS-PAGE gel and transferred onto nitrocellulose membrane (Hybond-C Extra; Amersham). Membranes were incubated in blocking buffer (TBS, 0.05% Tween and 5% milk powder) for 30 minutes at room temperature (RT), followed by incubation with the appropriate antibody overnight at 4°C. After several washes with TBS-T, membranes were incubated with appropriate peroxidase conjugated secondary antibodies in blocking buffer for one hour at RT followed by incubation with Super Signal West Dura Extended Duration Substrate (Pierce) and exposed to ECL films (Pierce).

For quantification of Western blots, intensity measurements of the protein bands were performed using ImageJ. For quantification of FUS-FMRP RNase +/- Western blots, values were corrected for FUS expression and normalized to the RNase negative condition (n = 2, one-way ANOVA). For quantification of Western blots of FUS-FMRP interaction with increasing RNA amounts, values were corrected for FUS expression and normalized to the 0 ng/µl RNA condition (n = 2, one-way ANOVA). For quantification of Western blots on zebrafish fractions, values were normalized against the corresponding non-injected control (NIC).

Primary motor neuron culture, transfection and immunocytochemistry

All animal care and use was in line with institutional, national and European legislation. Mice (C57BL/6) were purchased from Charles River. The ventral part of spinal cords of E13.5 mouse embryo's was dissected in phosphate buffered saline (PBS) and cut into small pieces. After incubation in 0.05% trypsin for 15 minutes at 37°C tissue was treated with DNAseI (Roche). Pieces were covered with 100 µl of 4% BSA and dissociated by pipetting. Dissociated cells were spun down at 2,000 rpm for 15 minutes in a 6% Optiprep gradient column. The motor neuron fraction was spun down at 800 rpm for 10 minutes to pellet cells. The cell pellet was dissolved in 1 ml of glia-conditioned medium (Neurobasal, 2 mM L-glutamine, 2% B27 which had been on glia cells for 24

hours) supplemented with 5% normal horse serum and GDNF, BDNF and CNTF (10 ng/ml each). Cells were plated on poly-D-ornithine and laminin coated coverslips. After 48 hours, cells were transfected using magnetic beads (Oz Biosciences) as described by Fallini et al. (2012) [20].

Primary motor neurons were fixed with 4% PFA for 15 min at RT, permeabilized with 0.1% Triton X-100 in PBS for 5 minutes at RT, blocked in PBS containing 2.5% BSA, and incubated with primary antibodies in BSA supplemented with 5% normal goat serum for 1 hour at RT. After several washes in PBS, cells were incubated with a mixture of the appropriate Alexa Fluor®-labeled secondary antibodies (Life Technologies) for 1 hour at RT. Then, cells were washed, counterstained with 4′,6′-diamidino-2-phenylindole (DAPI) (Sigma), washed extensively with PBS and mounted in DABCO.

Immunohistochemistry on human spinal cord samples

Consent for autopsy was obtained in concordance with institutional regulations. Patient and control details are included in Supplementary Table S3. For immunohistochemistry on human spinal cord samples, formalin-fixed paraffin-embedded 6 μm thick sections were deparaffinized in xylene and rinsed in graded ethanol (100%, 95%, 70%). Antigen retrieval was performed in citrate buffer (10 mM sodium citrate, pH 6.0) at 120°C for 10 minutes using a pressure cooker followed by incubation with a given primary antibody (rabbit anti-Upf1 1:200; rabbit anti-Caprin1 1:150; mouse anti-FMRP 1:150; rabbit anti-PABPC 1:50; rabbit anti-Dhx9 1:50). Due to antibody incompatibilities we were unable to perform double fluorescent-labeling for several interactors. For double immunohistochemistry, sections were incubated with Brightvision poly-alkaline phosphatase-anti-rabbit (Immunologic, Duiven, The Netherlands) for 30 min at RT, and washed with PBS. Sections were washed with Tris–HCl buffer (0.1 M, pH 8.2) to adjust the pH. Alkaline phosphatase activity was visualized with the alkaline phosphatase substrate kit I Vector Red (SK-5100, Vector laboratories Inc., CA, USA). To remove the first primary antibody, sections were incubated at 120°C in citrate buffer for 10 min. Incubation with rabbit anti-FUS (NB100-2599, 1:250) or anti-phospho-TDP-43 (1:4000) was performed overnight at 4°C. Alkaline phosphatase activity was visualized with the alkaline phosphatase substrate kit III Vector Blue (SK-5300, Vector laboratories Inc., CA, USA). Sections incubated without primary antibodies or with heat-inactivated primary antibodies were blank.

For double fluorescent-labeling, sections were blocked in 10% normal goat serum and 100 mM glycine in PBS for 40 minutes followed by incubation with the primary antibodies overnight at 4°C. Several PBS washes were followed by incubation with Alexa Fluor® 568-conjugated anti-rabbit and Alexa Fluor® 488-conjugated

anti-mouse (1:100, Molecular Probes, The Netherlands) for 2 h at RT. Subsequently, slides were incubated with Hoechst, followed by Sudan Black treatment (0.1% Sudan Black in ethanol for 10 minutes) and, after a short dip in 70% ethanol, mounted in 90% glycerol. Sections were analyzed using a laser scanning confocal microscope (Leica TCS Sp2, Wetzlar, Germany).

Zebrafish studies

Zebrafish were kept and maintained under standard conditions. Injections were performed at the one-cell stage. FUS WT, FUS R521C and mCherry-FMRP were subcloned into a pCS2+ vector and mRNA was transcribed from NotI linearized pCS2+ plasmid using SP6 polymerase and the mMESSAGE Machine kit (Ambion) followed by phenol-chloroform purification and ethanol precipitation. mRNAs were diluted in RNAse free water (with 1% phenol-red dye) at a concentration of 75-200 ng/μl and were pulse-injected using a pressure ejector. Touch-evoked escape response (TEER) was measured in developmentally normal zebrafish, as described previously [41]. In brief, TEER was assessed using a stereomicroscope at 72 hours post-fertilization (hpf). Zebrafish embryos were touched lightly at the tail using a closed pair of tweezers. Non-responding zebrafish were touched 2-3 times to confirm failure to respond.

For immunohistochemical analysis, zebrafish larvae were fixed in 4% PFA overnight at 4°C. Prior to staining, larvae were rinsed in PBS with 0.1% tween (PBS-T) and then permeabilized with collagenase A (1 mg/ml) for 45 min at RT. Larvae were washed three times in PBS-T and incubated with Alexa Fluor® 488-conjugated alpha-bungarotoxin in blocking buffer (2% BSA with 0.5% Triton-X100 in PBS) for 30 min. Larvae were subjected to several PBS-T washes (1 h each) and incubated with anti-SV2 antibody in blocking buffer at 4°C. Next, zebrafish larvae were subjected to several PBS-T washes (1.5 h each) and incubated in secondary antibody in blocking buffer for 4 h at RT. Finally, larvae were washed in PBS-T (1.5 h) and mounted in Mowiol (Sigma) for confocal imaging.

Image analysis and statistics

For Fmrp granule analysis, primary motor neurons were imaged using a Zeiss Axioskop2 microscope in combination with Axiovision SE64 software. For quantification of Fmrp granules, an automated method in ImageJ was designed. Per coverslip at least 10 transfected motor neurons were selected on basis of a healthy neuronal morphology. Pictures were taken at fixed locations over the axon (see Fig. S7a). The image was inverted in ImageJ, and a threshold was set at 130 using the "intermodes" threshold-mode. Then, the transfected

axon was selected using the selection brush tool (30 pixels) and the number of particles was quantified using "analyze particles" (size 3-30 pixel units, circularity 0.5-1). The total number of particles was divided by total axon area, resulting in a total number of granules/μm^2 axon. To test whether there was a significant difference in the average number of axonal Fmrp granules an ANOVA was performed.

For the zebrafish NMJ analysis, images were acquired using an Olympus Fluoview FV1000 confocal microscope using Olympus Fluoview software. Exposure was optimized for each image as to maintain a constant signal-to-noise ratio throughout image acquisition. Overlap between pre- and postsynapse was determined by loading z-stack projections of pre- and postsynapse images into the JACoP plugin for ImageJ [6]. Costes' automatic thresholding was applied to determine an optimal threshold for each channel. All statistical analyses were done in R (http://www.r-project.org) or Graphpad Prism 6 (La Jolla California, USA;http://www.graphpad.com).

Synaptosome isolation

Zebrafish embryos were collected at 72 hpf and yolk sacs were removed by triturating in ice-cold Ringer's salt solution (116 mM NaCl, 2.9 mM KCl, 1.8 mM $CaCl_2$, 5 mM HEPES) with 50 mM EDTA. Then, 200 to 500 embryo's were homogenized per condition in homogenization buffer (320 mM sucrose, 4 mM HEPES supplemented with protease inhibitors) using a tight glass douncer. Homogenates were centrifuged 4 times at 1,300 g for 10 minutes to pellet nuclei and the remaining supernatant was spun down at 15,000 g for 15 minutes to isolate synaptosomes.

Quantitative RT-PCR

Zebrafish embryos were collected as described above and 150-200 embryos per condition were homogenized and fractionated in homogenization buffer supplemented with 2% β-mercaptoethanol. TRIzol reagent was added to each fraction and total RNA was isolated. After DNAse treatment, cDNA was synthesized using superscript II reverse transcriptase (Life technologies) and oligo-dT primers. Quantitative real-time PCR was performed using SYBR green reagent (Roche) on a QuantStudio 6 Flex real time PCR system (Applied Biosystems) using primers against *Map1B* (FW: 5'- GGCAAAAGATTCTGCACCAC-3', RV: 5-' TGATGGGCTGTGTCTGAGAC-3'), *RhoA* (FW: 5'- GATGGAGCATGCGGGAAAAC -3', RV: 5-' CCTGCAGTATCCCACAGAGC-3'), *human-FUS* (FW: 5'- ATGGACAGCAGAACCAGTAC-3', RV: 5-' GGTCTTGATTGCCATAACCG-3'), *human-FMRP* (FW: 5'- GCTCCAACAGAGGAAGAGAGG-3', RV: 5-'

ATCTGTTCGGGAGTGATCGT -3'), and mRNA levels were normalized to *Gapdh* (FW: 5'-GTGGTGCAAAGAGAGTCAT-3', RV: 5-' -3') and *β-actin* (FW: 5'- CTCCCCTGAATCCCAAAGCC -3', RV: 5-' GAGAGAGCACAGCCTGGATG-3') housekeeping genes. Fractions were normalized to their respective inputs and fold changes were then calculated compared to non-injected controls using standard comparative ΔΔCT methods. Samples from three experiments were amplified in triplicate and results were statically analyzed using Student's t-test.

Polysomal fractionations

Polysomal fractionations from N2A cells were performed using a protocol adapted from Gismondi et al., 2014 [27]. $4x10^6$ of N2A cells were plated on 15-cm plates. After 72 h, cells were treated with 100 μg/ml cycloheximide for 5 minutes, washed twice with PBS supplemented with 100 μg/ml cycloheximide, and then lysed with 400 μl of TNM Lysis Buffer (10 mM Tris pH 7.5; 10 mM NaCl; 10 mM $MgCl_2$; 1% Triton X100; DTT 10mM) supplemented with 100 μg/ml cycloheximide, protease inhibitor cocktail (Roche) and RiboLock RNase Inhibitor (Thermo Scientific) and transferred into a microcentrifuge tube. Lysates were incubated on ice for 10 minutes and then centrifuged at 16,000 g at 4°C. The supernatant was layered onto a 15–50% (w/v) sucrose gradient (containing 10mM Tris-HCl pH 7.5, 100 mM NaCl and 10 mM $MgCl_2$) and centrifuged in a Beckman SW41 rotor for 90 minutes at 37,000 rpm at 4°C. Fractions were collected with a Bio-logic LP, Model 2110 Fraction Collector (Biorad). Proteins from each polysomal fraction were TCA precipitated.
