# Supplementary material for: Comparative interactomics analysis of different ALS-associated proteins identifies converging molecular pathways
Source: Acta Neuropathol. 2016 May 10;132:175–96. doi: 10.1007/s00401-016-1575-8 (PMC4947123; doi:10.1007/s00401-016-1575-8)
Supplement: Supplementary file 2 — Supplementary material 2 (PDF 215 kb) [file 401_2016_1575_MOESM2_ESM.pdf]

Supplementary Tables

Comparative interactomics analysis of different ALS-associated proteins identifies converging molecular pathways

Anna M. Blokhuis, Max Koppers, Ewout J.N. Groen, Dianne M.A. van den Heuvel, Stefano Dini Modigliani, Jasper J. Anink, Katsumi Fumoto, Femke van Diggelen, Anne Snelting, Peter Sodaar, Bert M. Verheijen, Jeroen A.A. Demmers, Jan H. Veldink, Eleonora Aronica, Irene Bozzoni, Jeroen den Hertog, Leonard H. van den Berg and R. Jeroen Pasterkamp

Corresponding author:

R. J. Pasterkamp

Department of Translational Neuroscience, Brain Center Rudolf Magnus, University Medical Center Utrecht, Utrecht, The Netherlands

r.j.pasterkamp@umcutrecht.nl

Includes:

Supplementary Table S1a-f

Supplementary Table S2

Supplementary Table S1a: ATXN2 interactors and GO analysis

DNA/RNA helicases	Elavl proteins	EIFs	FXR proteins	hnRNPs	Other RNA/DNA binding proteins		Ribosomal proteins	snRNPs	Others	Splicing factors
Ddx1	Elavl1	Eif2c2	Fmr1	Hnrnpa0	Alyref	Msi1	Rpl11	Snrpa	4930550L24Rik	Sf3b2
Ddx21	Elavl2	Eif2s1	Fxr1	Hnrnpa1	Atxn2l	Msi2	Rplp0	Snrpa1	Ahnak	Sf3b3
Ddx3x	Elavl3	Eif3a	Fxr2	Hnrnpa2b1	C1qbp	Myef2	Rps10		Alg13	Sfpq
Ddx5	Elavl4	Eif3b		Hnrnpa3	Caprin1	Ncbp1	Rps12		Cad	Srsf10
Ddx6		Eif3c		Hnrnpab	Carm1	Ncl	Rps13		Cct5	Srsf3
Dhx15		Eif3d		Hnrnpc	Casc3	Nufip2	Rps14		Dynll1	Srsf7
Dhx29		Eif3g		Hnrnpk	Cpsf1	Pabpc1	Rps15a		Fam120c	Tra2a
Dhx30		Eif3h		Hnrnpl	Cpsf2	Pabpc4	Rps16		Fam98a	Tra2b
Dhx36		Eif3i		Hnrnpm	Csde1	Pcbp2	Rps17		Gys1	
Dhx9		Eif3k		Hnrnpr	D10Wsu52e	Pcbp3	Rps18		Iap	
		Eif3l		Hnrnpu	FAM120A	Prkra	Rps19		Kpnb1	
		Eif3m		Hnrnpul2	Fam195a	Ptbp1	Rps2		Lsm12	
		Eif4e		Hnrpdl	G3bp1	Pum1	Rps20		Map1b	
		Eif4g1			G3bp2	Pum2	Rps21		Nxf7	
		Eif4g3			Gemin5	Pura	Rps25		Pgam5	
					Gm10704	Purb	Rps27		Ppp1ca	
					Gnb2l1	Rbm8a	Rps28		Ppp2cb	
					Igf2bp3	Strap	Rps3		Ppp2r1a	
					Ilf3	Syncrip	Rps3a		Prrc2a	
					Larp1	Tdrd3	Rps4x		Prrc2c	
					Larp4	Top3b	Rps6		Timm50	
					Larp4b	Upf1	Rps7		Tox4	
					Lin28a	Upf2	Rps8		Trim28	
					Magoh	Ybx1	Rps9		Ywhae	
					Matr3	Zc3hav1	Rpsa		Ywhaz	
					Mov10	Zfr	Rrbp1			
							Ubap2l			

Supplementary Table S1a: ATXN2 interactors and GO analysis (cont.)

**GO biological process complete ATXN2**	**P-value**
posttranscriptional regulation of gene expression (GO:0010608)	1.82E-41
RNA processing (GO:0006396)	5.10E-36
translation (GO:0006412)	2.98E-32
gene expression (GO:0010467)	8.05E-31
RNA splicing (GO:0008380)	4.33E-23
translational initiation (GO:0006413)	2.93E-20
establishment of RNA localization (GO:0051236)	1.61E-11
RNA transport (GO:0050658)	1.61E-11
regulation of mRNA stability (GO:0043488)	1.83E-09
rRNA processing (GO:0006364)	4.74E-05
stress granule assembly (GO:0034063)	6.89E-03

**GO cellular component complete ATXN2**	**P-value**
ribonucleoprotein complex (GO:0030529)	5.22E-78
ribosome (GO:0005840)	7.73E-29
cytoplasmic ribonucleoprotein granule (GO:0036464)	2.07E-24
cytoplasmic stress granule (GO:0010494)	1.07E-23
spliceosomal complex (GO:0005681)	1.22E-17
eukaryotic translation initiation factor 3 complex (GO:0005852)	3.34E-16
polysome (GO:0005844)	8.03E-12
CRD-mediated mRNA stability complex (GO:0070937)	1.17E-06
exon-exon junction complex (GO:0035145)	1.01E-05
cytoplasmic mRNA processing body (GO:0000932)	3.56E-03

Supplementary Table S1b: C9ORF72 interactors and GO analysis

C9orf72 homolog	MICOS	Mitochondrial proteins	Chaperones	Other
Smcr8	Chchd3	Atad3	Cct2	Ahsa1
	Immt	Atp5a1	Cct5	Asz1
		Atp5c1	Cct7	Atp1a1
		Dnaja1	Dnaja2	Atp2a2
		Pdha1	Dnajb11	Bdh1
		Pdhb	Dnajb12	Cad
		Pdhx	Tcp1	Ckap4
		Sdha		Eef2
		Slc25a12		Far1
		Slc25a3		Hnrnpf
		Slc25a4		Iap
		Slc25a5		Map1b
		Timm50		Ncl
		Vdac3		Ppp1ca
		Wdr41		Rplp0
				Rtn4
				Slc16a1
				Tox4

Supplementary Table S1b: C9ORF72 interactors and GO analysis (cont.)

GO biological process complete C9ORF72	P-value
protein folding (GO:0006457)	1.50E-04
mitochondrial membrane organization (GO:0007006)	4.12E-02
transport (GO:0006810)	1.47E-02

GO cellular component complete C9ORF72	P-value
myelin sheath (GO:0043209)	1.53E-15
mitochondrial inner membrane (GO:0005743)	2.40E-10
chaperonin-containing T-complex (GO:0005832)	3.73E-06

Supplementary Table S1c: FUS interactors and GO analysis

DNA/RNA helicases	Elavl proteins	EIFs	FXR proteins	hnRNPs	Other RNA/DNA binding proteins		Ribosomal proteins	snRNPs	Others	Histone proteins	RFs	Splicing factors
Ddx1	Elavl1	Eif3b	Fmr1	Hnrnpa0	Alyref	Pabpc4	Gm9493	Eftud2 (U5)	Erh	H2afx	Rfc1	Sf3a1 (U11)
Ddx21	Elavl2	Eif3c	Fxr1	Hnrnpa1	Atxn2l	Parp1	Rpl11	Snrnp200 (U5)	Fam98a	Hist1h1a	Rfc2	Sf3b1 (U12)
Ddx3x	Elavl3		Fxr2	Hnrnpa2b1	Bclaf1	Parp2	Rpl12	Snrpa (U1)	Luzp4	Hist1h2al	Rfc3	Sf3b2
Ddx5	Elavl4			Hnrnpa3	Caprin1	Pnkp	Rplp0	Snrpa1 (U2)	Iap	Hist1h2bp	Rfc4	Sf3b3
Dhx15				Hnrnpk	Cdkn2aip	Polb	Rps14	Snrpb	Lgals3bp	Hist1h3b	Rfc5	Sfpq
Dhx30				Hnrnpl	Chd1l	Prpf19	Rps15a	Thrap3	Trim28	Hist1h4a		Srsf10
Dhx36				Hnrnpm	D10Wsu52e	Prpf8	Rps16					Srsf7
Dhx9				Hnrnpr	G3bp1	Prrc2c	Rps17					Tra2a
				Hnrnpu	G3bp2	Ptbp1	Rps18					Tra2b
				Hnrnpul1	Gm10704	Raly	Rps19					
				Hnrpdl	Gnb2l1	Rbm14	Rps25					
					Hnrnpc	Rpa1	Rps3					
					Khdrbs1	Ssrp1	Rps3a					
					Larp1	Supt16	Rps4x					
					Lig3	Syncrip	Rps8					
					Magoh	Tmpo	Rpsa					
					Matr3	Top1						
					Mov10	Top2a						
					Myef2	Upf1						
					Ncbp1	Xrcc1						
					Ncl	Xrcc5						
					Nono	Xrcc6						
					Nufip2	Xrn2						
					Pabpc1	Zc3h11a						

Supplementary Table S1c: FUS interactors and GO analysis (cont.)

**GO cellular component complete FUS**	**P-value**
ribonucleoprotein complex (GO:0030529)	1.48E-63
spliceosomal complex (GO:0005681)	2.40E-29
ribonucleoprotein granule (GO:0035770)	3.36E-17
cytosolic ribosome (GO:0022626)	1.31E-16
chromosome (GO:0005694)	3.51E-12
cytoplasmic stress granule (GO:0010494)	1.72E-08
DNA replication factor C complex (GO:0005663)	1.32E-07
polysome (GO:0005844)	2.71E-06
paraspeckles (GO:0042382)	6.48E-03
CRD-mediated mRNA stability complex (GO:0070937)	6.48E-03

**GO biological process complete FUS**	**P-value**
RNA processing (GO:0006396)	2.18E-38
RNA splicing (GO:0008380)	1.65E-32
DNA repair (GO:0006281)	7.69E-12
regulation of mRNA stability (GO:0043488)	4.58E-09
RNA transport (GO:0050658)	7.01E-09
translation (GO:0006412)	2.47E-07
DNA replication (GO:0006260)	6.91E-07
ribosomal small subunit biogenesis (GO:0042274)	9.28E-07

Supplementary Table S1d: OPTN interactors and GO analysis

Kinases	Motor proteins	Autophagy /NFkB signaling	14-3-3-proteins	ERAD	Others
Csnk2a1	Dynll1	Ubc	Ywhab	Nploc4	2410002F23Rik
Csnk2a2	Dynll2	Sqstm1	Ywhae		Dhx15
		Tax1bp1	Ywhag		Flot1
		Tbk1	Ywhah		Iap
		Vcp	Ywhaq		Prkar1a
		Rnf31	Ywhaz		Rpn1
					Tbc1d15
					Tmem66
					Trim28
					Wrnip1

GO biological process complete OPTN	P-value
intracellular protein transport (GO:0006886)	1.32E-04
cellular protein localization (GO:0034613)	1.46E-03

GO cellular component complete OPTN	P-value
membrane-bounded organelle (GO:0043227)	2.68E-02
vesicle (GO:0031982)	5.70E-03
protein kinase CK2 complex (GO:0005956)	8.47E-03

Supplementary Table S1e: TDP-43 interactors and GO analysis

DNA/RNA helicases	Elavl proteins	EIFs	FXR proteins	hnRNPs	Other RNA/DNA binding proteins		Ribosomal proteins	snRNPs	Others	Splicing factors
Ddx1	Elavl1	Eif2s1	Fmr1	Hnrnpa0	Alyref	Nop58	Rpl11	Coil	Iap	Sf3a1 (U11)
Ddx21	Elavl2	Eif2s3x	Fxr1	Hnrnpa1	Atxn2l	Pabpc1	Rpl38	Eftud2 (U5)	Luzp4	Sf3b1 (U12)
Ddx39a	Elavl3	Eif3a	Fxr2	Hnrnpa2b1	Caprin1	Pabpc4	Rplp0	Snrnp200 (U5)	Prrc2c	Sf3b2
Ddx39b	Elavl4	Eif3b		Hnrnpa3	Cdc5l	Pabpn1	Rps14	Snrpa1 (U2)		Sf3b3
Ddx3x		Eif3c		Hnrnpab	Celf1	Pcbp2	Rps16			Sfpq
Ddx5		Eif3e		Hnrnpc	Csde1	Pcbp3	Rps17			Snrpb
Ddx6		Eif3f		Hnrnpd	D10Wsu52e	Poldip3	Rps18			Srsf10
Dhx15		Eif3g		Hnrnpf	FAM120A	Prpf19	Rps19			Srsf3
Dhx29		Eif3h		Hnrnph1	Fbl	Prpf8	Rps20			Srsf7
Dhx30		Eif3i		Hnrnpk	G3bp1	Ptbp1	Rps25			Tra2a
Dhx36		Eif3l		Hnrnpl	Gar1	Pura	Rps3			Tra2b
Dhx9		Eif4a1		Hnrnpm	Gm10704	Raly	Rps3a			
		Eif4a3		Hnrnpr	Gnb2l1	Rbm14	Rps4x			
		Eif4g1		Hnrnpu	Ilf2	Rbm8a	Rps7			
				Hnrnpul1	Ilf3	Rbmxl1	Rps8			
				Hnrnpul2	Khdrbs1	Srrt	Rpsa			
				Hnrpdl	Larp1	Ssb				
					Larp4	Strap				
					Magoh	Syncrip				
					Matr3	Upf1				
					Mov10	Ybx1				
					Msi2	Ybx3				
					Myef2	Zc3h11a				
					Ncl	Zc3h14				
					Nhp2l1	Zc3hav1				
					Nolc1	Zfr				
					Nono	Znf326				
					Nop56					

Supplementary Table S1e: TDP-43 interactors and GO analysis (cont.)

**GO biological process complete TDP-43**	**P-value**
RNA processing (GO:0006396)	8.36E-59
gene expression (GO:0010467)	6.91E-51
RNA splicing (GO:0008380)	1.63E-45
posttranscriptional regulation of gene expression (GO:0010608)	8.57E-34
translation (GO:0006412)	7.48E-26
translational initiation (GO:0006413)	8.98E-18
regulation of mRNA stability (GO:0043488)	2.79E-13
RNA transport (GO:0050658)	2.56E-12
ncRNA processing (GO:0034470)	6.33E-07
rRNA processing (GO:0006364)	8.78E-07
RNA export from nucleus (GO:0006405)	1.17E-04
ribonucleoprotein complex export from nucleus (GO:0071426)	2.10E-04

**GO cellular component complete TDP-43**	**P-value**
ribonucleoprotein complex (GO:0030529)	7.92E-98
spliceosomal complex (GO:0005681)	2.38E-42
ribonucleoprotein granule (GO:0035770)	4.67E-19
ribosome (GO:0005840)	3.30E-15
eukaryotic translation initiation factor 3 complex (GO:0005852)	1.07E-14
cytoplasmic stress granule (GO:0010494)	3.21E-13
eukaryotic translation initiation factor 3 complex, eIF3m (GO:0071541)	2.11E-08
CRD-mediated mRNA stability complex (GO:0070937)	6.34E-07
polysome (GO:0005844)	6.72E-06
box C/D snoRNP complex (GO:0031428)	8.74E-05
exon-exon junction complex (GO:0035145)	2.43E-04
Cajal body (GO:0015030)	1.49E-03
histone pre-mRNA 3'end processing complex (GO:0071204)	9.58E-03
paraspeckles (GO:0042382)	9.58E-03

Supplementary Table S1f: UBQLN2 interactors and GO analysis

14-3-3 proteins	Autophagy /NFkB signaling	Bat3 complex	E3 ubiquitin ligases	Endo/ lysosomal trafficking	ERAD	ER-Golgi	Kinases	Proteasome proteins	Others	
Ywhab	Nbr1	Asna1	Hltf	Atp6v0a1	Faf2	Arf1	Csnk2a1	Adrm1	Ankrd13a	Lgals8
Ywhae	Optn	Bag6	Itch	Atp6v0d1	Nploc4	Get4	Csnk2b	Psma3	Atxn10	Nvl
Ywhag	Sqstm1	Ubl4a	Trim32	Atp6v1a	Ufd1l	Rab1	Gsk3b	Psma5	Bag2	Pcm1
Ywhah	Tax1bp1			Atp6v1e1	Vcp	Rab6a		Psma6	Calcoco1	Pdcd6
Ywhaz	Tbk1			Cltc		Rap1		Psma7	Dnaja2	Peg10
	Tnip1			Hap1				Psmb1	Eef2	Ppia
	Tollip			Pdcd6ip				Psmb2	Eno1	Prkar1a
	Ub			Rab7a				Psmb3	Fam115a	Ret
	Ubqln1			Stam				Psmb5	Fth1	Rtl1
	Vcp			Tbc1d15				Psmb6	Ftl	Sdcbp
				Tbc1d17				Psmc1	Gapdh	Stmn3
				Tom1				Psmc2	Iap	Stom
								Psmc3	Keap1	Tmem66
								Psmc4	Kpnb1	Tprgl
								Psmc5		Vim
								Psmc6		Wbp2
								Psmd1		Wrnip1
								Psmd11		
								Psmd12		
								Psmd13		
								Psmd2		
								Psmd3		
								Psmd6		
								Psmd7		

Supplementary Table S1f: UBQLN2 interactors and GO analysis (cont.)

**GO biological process complete UBQLN2**	**P-value**
cellular protein catabolic process (GO:0044257)	8.90E-27
proteasome-mediated ubiquitin-dependent protein catabolic process (GO:0043161)	5.49E-19
ERAD pathway (GO:0036503)	5.79E-14
response to endoplasmic reticulum stress (GO:0034976)	1.74E-10
cellular protein localization (GO:0034613)	8.10E-08

**GO cellular component complete UBQLN2**	**P-value**
proteasome complex (GO:0000502)	3.34E-46
extracellular vesicle (GO:1903561)	2.51E-25
VCP-NPL4-UFD1 AAA ATPase complex (GO:0034098)	5.92E-05
ER membrane insertion complex (GO:0072379)	3.85E-04
BAT3 complex (GO:0071818)	3.85E-04

Supplementary Table S2: Overlapping proteins FUS, TDP-43 and ATXN2 and OPTN and UBLQN2

Overlapping proteins FUS, TDP-43 and ATXN2

Elavl proteins	EIFs	FXR proteins	hnRNPs	Other RNA/DNA binding proteins	Ribosomal proteins	snRNPs	Splicing factors	Others
Elavl1	Eif3b	Fmr1	Hnrnpa0	Alyref	Rps7	Snrpa1	Sf3b2	Iap
Elavl2	Eif3c	Fxr1	Hnrnpa1	Atxn2l	Rpl11		Sf3b3	
Elavl3		Fxr2	Hnrnpa2b1	Caprin1	Rplp0		Sfpq	
Elavl4			Hnrnpa3	D10Wsu52e	Rps14		Srsf10	
			Hnrnpk	G3bp1	Rps16		Srsf7	
			Hnrnpl	Gm10704	Rps17		Tra2a	
			Hnrnpm	Gnb2l1	Rps18		Tra2b	
			Hnrnpr	Larp1	Rps19			
			Hnrnpu	Magoh	Rps25			
			Hnrpdl	Matr3	Rps3			
				Mov10	Rps3a			
				Myef2	Rps4x			
				Ncl	Rps8			
				Pabpc1	Rpsa			
				Pabpc4				
				Prrc2c				
				Ptbp1				
				Syncrip				
				Upf1				

Supplementary Table S2: Overlapping proteins FUS, TDP-43 and ATXN2 and OPTN and UBLQN2 (cont.)

Overlapping proteins OPTN and UBQLN2

14-3-3 proteins	Autophagy/NFkB signaling	Endo-lysosomal trafficking	ERAD	Kinases	Others
Ywhab	Sqstm1	Tbc1d15	Nploc4	Csnk2a1	Iap
Ywhae	Tax1bp1				Prkar1a
Ywhag	Tbk1				Wrnip1
Ywhah	Vcp				Tmem66
Ywhaz					
